# Metabolic modeling of host–microbe interactions for therapeutics in colorectal cancer

**DOI:** 10.1038/s41540-021-00210-9

**Published:** 2022-01-19

**Authors:** Prerna Bhalla, Raghunathan Rengaswamy, Devarajan Karunagaran, G. K. Suraishkumar, Swagatika Sahoo

**Affiliations:** 1 Department of Biotechnology, Bhupat and Jyoti Mehta School of Biosciences Building, Indian Institute of Technology Madras, Chennai, 600036 India; 2 Department of Chemical Engineering, Indian Institute of Technology Madras, Chennai, 600036 India; 3 Initiative for Biological Systems Engineering, Indian Institute of Technology Madras, Chennai, 600036 India

**Keywords:** Multicellular systems, Biochemical networks, Systems analysis, Computer modelling

## Abstract

The onset of colorectal cancer (CRC) is often attributed to gut bacterial dysbiosis, and thus gut microbiota are highly relevant in devising treatment strategies. Certain gut microbes, like *Enterococcus spp*., exhibit remarkable anti-neoplastic and probiotic properties, which can aid in silver nanoparticle (AgNPs) induced reactive oxygen species (ROS)-based CRC treatment. However, the effects of AgNPs on gut microbial metabolism have not been reported thus far. In this study, a detailed systems-level understanding of ROS metabolism in *Enterococcus durans* (*E. durans*), a representative gut microbe, was gained using constraint-based modeling, wherein, the critical association between ROS and folate metabolism was established. Experimental studies involving low AgNP concentration treatment of *E. durans* cultures confirmed these modeling predictions (an increased extracellular folate concentration by 52%, at the 9^th^ h of microbial growth, was observed). Besides, the computational studies established various metabolic pathways involving amino acids, energy metabolites, nucleotides, and SCFAs as the key players in elevating folate levels on ROS exposure. The anti-cancer potential of *E. durans* was also studied through MTT analysis of HCT 116 cells treated with microbial culture (AgNP treated) supernatant. A decrease in cell viability by 19% implicated the role of microbial metabolites (primarily folate) in causing cell death. The genome-scale modeling approach was then extended to extensively model CRC metabolism, as well as CRC–*E. durans* interactions in the context of CRC treatment, using tissue-specific metabolic models of CRC and healthy colon. These findings on further validation can facilitate the development of robust and effective cancer therapy.

## Introduction

**Fig. 1:**
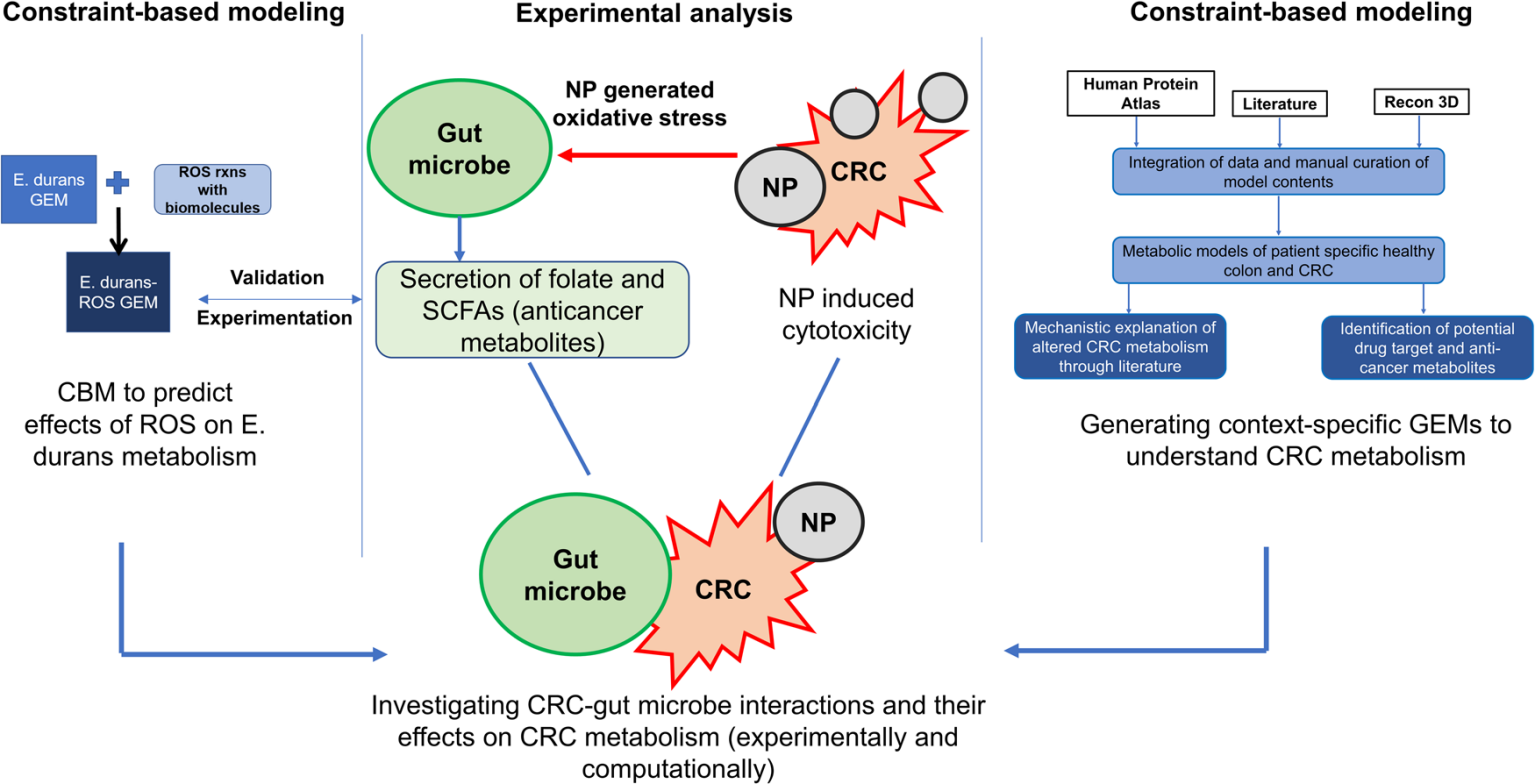
An overview of the direct and indirect effects of AgNPs mediated oxidative stress on gut bacterial metabolism in treatment and management of CRC. AgNPs are known to mediate their direct cytotoxic effects through increased generation of reactive species inside the target cells. Besides, the AgNP-generated oxidative stress can also affect the cellular metabolism and certain pathways of interest (in this case, the anti-cancer metabolites) thereby enhancing indirect cytotoxicity. Gut microbe-CRC interactions can further assist in understanding the role of gut bacterial metabolites (indirect effects of ROS) on CRC metabolism.

The onset of colorectal cancer (CRC) has been associated with various extrinsic factors such as infection, unhealthy diet, and lifestyle^1–3^, which often result in gut microbiota dysbiosis—a clinical disorder characterized by perturbation in the composition and function of the healthy gut microbiota^4,5^. In the state of homeostasis, the gut bacteria-derived metabolites synthesize essential nutrients and compounds^6,7^, some of which have demonstrated remarkable anti-neoplastic properties^8^. For instance, Enterococcal peptides obtained from clinical strains of *Enterococcus* genus (a representative gut microbe) exhibit anti-proliferative effects against colorectal adenocarcinoma and other cancer types^9^. Furthermore, *Enterococcus durans* was shown to produce butyrate, which is anti-inflammatory in nature and is required to maintain the integrity of intestinal epithelium^10^. Such studies have highlighted the relevance of gut microbes in devising effective, novel cancer treatment therapies, as in the case of nanoparticle-based CRC treatment (Fig. 1).

The transformation of a healthy colon cell into a cancerous cell type is a complex event, which disrupts cell characteristics at metabolic, signaling, and regulatory levels^11,12^, that have been captured through various in vitro and in vivo experiments. Moreover, with the advent of constraint-based modeling (CBM), genome-scale metabolic models (GSMMs) too have aimed at investigating the re-programmed metabolism for devising novel therapeutic strategies^13,14^. To illustrate, context-specific metabolic models of colon and CRC cell types have been developed to understand the aberrant metabolism in CRC, as well as deciphering drug targets and biomarkers for cancer diagnostics. For instance, one such experimental and computational study discovered the FUT9 gene as a crucial promoter of advanced stage colon cancer^15^. Despite these computational advances, there have been limited computational studies to understand the contribution of reactive species generation (known to exert cytotoxic effects in cancer cells) and gut microbiome secreted metabolites towards CRC metabolism and treatment. Therefore, it would be interesting to investigate and model the role of gut bacteria secreted metabolites in cancer cell killing.

Here we present a computational and experimental study that reveals the effects of AgNP-mediated reactive species generation on gut bacterial metabolism. The effects of reactive oxygen species (ROS) were extensively modeled using the GSMM of *E. durans*. These computational findings were supported through experiments. Furthermore, the complexity and systems-level understanding of the integral metabolic interactions between gut microbe and host (i.e., colon and CRC cells) was gained using tissue-specific metabolic models. These models were developed from proteomics and literature-based data sources, representing healthy colon and CRC conditions. In addition, we have reported the differences between the CRC vs. healthy colon models in terms of their biomass values and attempted at elucidating the metabolic alterations behind aberrant CRC metabolism using genome-scale metabolic modeling.

## Results

### AgNP-mediated oxidative stress (ROS) modulated gut microbial metabolism

**Fig. 2:**
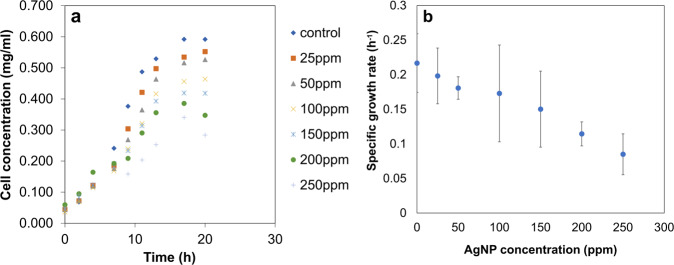
Variation of *E. durans* growth with different concentrations of AgNPs. Exposure of bacterial cultures to higher concentrations of the nanoparticles results in **a**) excessive cell death and **b**) reduced specific growth rates (*p*-value = 0.0201). Values are expressed as mean ± SD, *n* = 4.

**Fig. 3:**
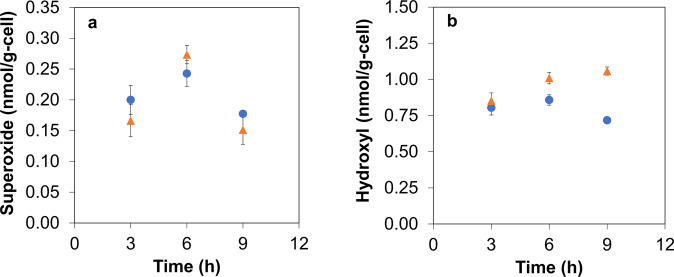
Reactive species time profile in the absence and presence of AgNP generated oxidative stress. ( indicates control culture;  indicates culture treated with 25 ppm AgNP). **a** The superoxide radical levels increased during the mid-log phase (6th h) of growth (on exposure to AgNPs), and then dropped, when compared to control (*p*-value = 0.0419). **b** The hydroxyl radical concentration in the presence of a lower concentration of AgNP, showed increased levels at 9th h when compared to the control (*p*-value = 0.0357). Values are expressed as mean ± SD, *n* = 3.

The AgNPs used in this study were characterized for their physicochemical properties (Supplementary Fig. 1). To study the effects of AgNPs on the growth and cell viability of *E. durans*, the bacterial cultures were exposed to different AgNP concentrations (Fig. 2). The specific growth rate (µ) for the cultures treated with the lowest AgNP concentration (25 ppm) was 0.198 ± 0.03 h^−1^, which was 8% lower compared to the control. Further, the highest AgNP concentration (250 ppm) reduced µ to 0.08 ± 0.016 h^−1^, thereby indicating the deleterious effects of nanoparticles at higher concentrations. Thus, the lowest concentration of AgNPs (25 ppm) exhibited no major detrimental effects on microbial viability. Further, the effects of AgNPs at such low concentrations were quantified experimentally on ROS generation (superoxide and hydroxyl radicals) (Fig. 3a, b). At 6th h (mid-log phase) of AgNP-treated bacterial culture growth, the specific intracellular superoxide concentration increased by 13% (0.273 ± 0.01 nmol/g-cell) compared to control. Similarly, specific intracellular hydroxyl radical levels showed a 48% increase (1.057 ± 0.02 nmol/g-cell, at 9th h) in the late-log phase, compared to control.

Nevertheless, the AgNP-induced intracellular ROS generation could have altered microbial metabolism. This altered state of metabolism was captured using the CBM approach, which is a preferred computational approach for studying overall metabolic characteristics and metabolite systems profiling^16^, as experimental studies cannot capture all the metabolic complexities simultaneously. The metabolic effects of ROS (oxidative stress) on *E. durans* were computationally captured for the first time in this study, wherein CBM of *E. durans* was used to identify metabolic consequences associated with increased generation of reactive species (ROS/RNS) within the microbial system. Genome-scale metabolic model (GSMM) of *E. durans* (*E. durans* ATCC 6056) was downloaded from virtual human metabolic (VMH)^17^, and an extensive manual curation of the model was performed using scientific literature to incorporate relevant reactive species reactions to generate expanded *E. durans* model with ROS reactions (Supplementary Data 1 and Supplementary Table 1). The ROS expanded metabolic model was then constrained as per the MRS medium composition (Supplementary Data 1).

**Fig. 4:**
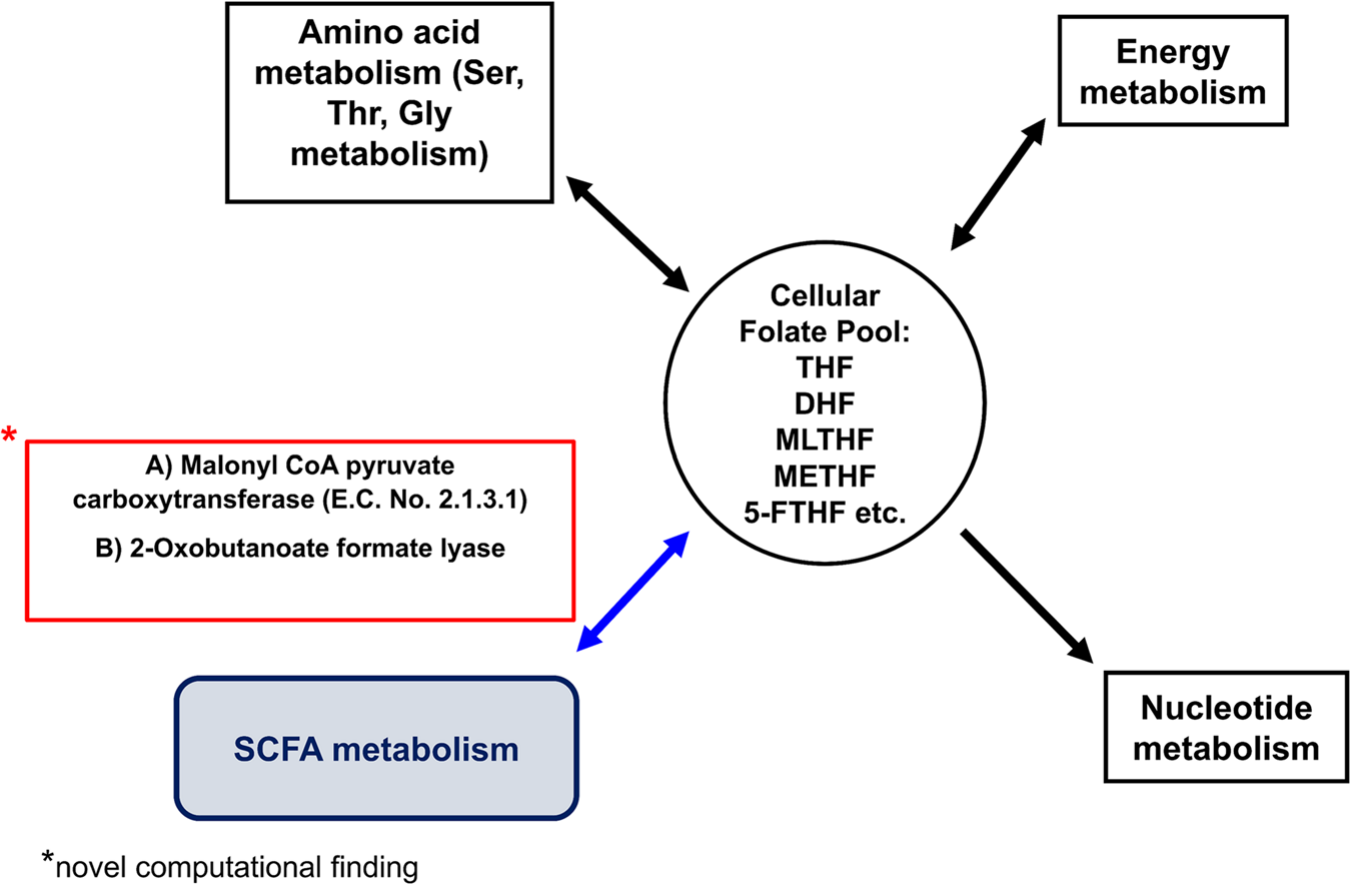
Folate metabolism and its association with different central metabolic pathways. The metabolic pathways (viz., amino acid, energy and nucleotide metabolism), were influenced by folate metabolism as predicted by the model. The link between SCFA metabolism and folic acid metabolic pathways is a novel model prediction that needs to be confirmed experimentally.

Flux variability analysis (FVA)^18^ was then implemented to capture the relative changes in the network fluxes before and after the addition of ROS reactions. Upon calculating flux span ratio (FSr) for the original and ROS expanded models (detail in the Methods section), seven reactions- most of them associated with folate metabolism, showed heightened fluxes in the expanded ROS model. These reactions were catalyzed by: dihydrofolate reductase (EC:1.5.1.3), methenyltetrahydrofolate cyclohydrolase (EC:3.5.4.9), formate-tetrahydrofolate ligase (EC:6.3.4.3). In addition, minNorm analysis indicated that these seven reactions, in turn, influenced major metabolic pathways, i.e., amino acid/peptide metabolism, nucleotide metabolism, carbohydrate metabolism (pyruvate metabolism, glycolysis/gluconeogenesis), and energy metabolism, as elucidated in Fig. 4 (details in Discussion and Supplementary Table 2). One of the most important and novel CBM predictions highlighted the association between folic acid derivatives (cellular folate pool) and SCFA (propionate) metabolism through the enzymes-malonyl CoA pyruvate carboxytransferase (EC:2.1.3.1) and 2-oxobutanoate formate lyase, as mentioned in Supplementary Table 2.

**Fig. 5:**
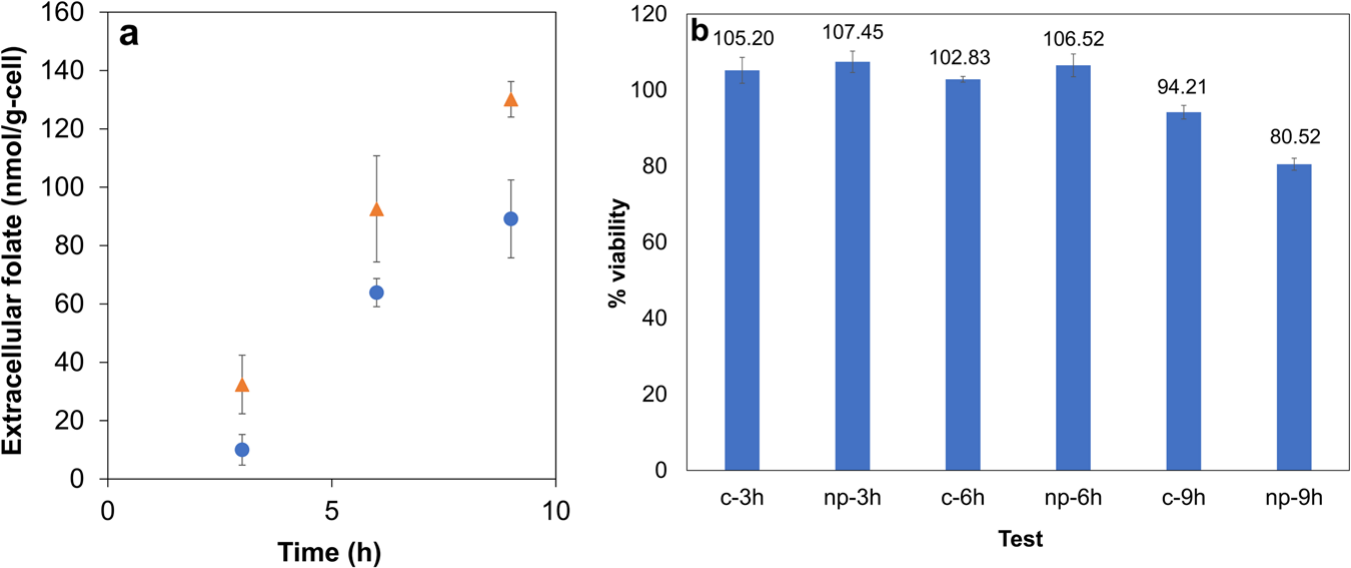
Effects of AgNPs on folate secretion, and role of latter in affecting HCT116 viability. ( indicates control culture;  indicates culture treated with 25 ppm AgNP). **a** The extracellular folic acid concentrations increased on exposure to AgNPs. This increase could be an outcome of the oxidative stress induced in the organism, which in turn impact folate related metabolic pathways. **b** MTT analysis of HCT116 treated with bacterial supernatants from cultures exposed to silver nanoparticles and control. Reduction in viability was observed for cells treated with 9th h supernatants (*p*-value = 0.0031), the time point corresponding to maximum concentration. Values are expressed as mean ± SD, *n* = 3.

Based on the modeling predictions, the effects of ROS on folate levels were examined experimentally. Interestingly, the findings showed AgNP-induced oxidative stress also elevated both intracellular (Supplementary Fig. 2) and extracellular (Fig. 5a) microbial folate levels, as revealed by HPLC analysis. At 9th h of AgNP-treated microbial growth, maximum specific extracellular folate was 52% higher compared to control (128.84 ± 0.16 nmol/g-cell).

### *E. durans*-secreted metabolites, specifically folate, affected CRC cell viability

Previous studies have reported that many microbe-secreted metabolites exhibit anticancer properties^8,19^. Folic acid is one such metabolite, which is involved in CRC carcinogenesis^20,21^. However, for the very first time, we were able to identify the cytotoxic effects of folic acid (at a critical concentration of 0.5 µM) on HCT 116 cells (Supplementary Fig. 3). Moreover, the role of various metabolites produced by *E. durans* in the context of nanoparticles-based targeting of CRC was studied. To do so, HCT 116 cells were treated with crude bacterial supernatant obtained from AgNP-treated bacterial culture and an MTT viability assay was performed. It was found that AgNP (25 ppm) treated bacterial culture supernatant exerted cytotoxic effects on cancer cells. The viability of the cancer cells treated with 9th h supernatant from nanoparticle treated cultures was reduced by 19% as compared to control (Fig. 5b). Interestingly, this was also the time point corresponding to the release of the optimal cytotoxic concentration of extracellular folate secreted in the culture (Fig. 5a). As microbe secreted metabolites have the potential to affect cancer cell viability, it was crucial to understand the metabolic interrelationships between the *E. durans* and the host cell (i.e., healthy colon and cancer colon cell types) in detail.

### Context-specific metabolic models of CRC and CRC-microbe metabolic interactions facilitated understanding metabolic complexities of CRC

Tissue-specific metabolic models of healthy colon and CRC cells were developed from Recon 3D^22^. The generic human metabolic network was constrained using proteomic data obtained from the Human Protein Atlas (HPA; Version: 19.1)^23^, as well as gene regulation data (genes specifically upregulated in colon cancer) from the literature^24^. The multi-omics data was then integrated using iMAT algorithm^25^. The data-constrained models were further conditioned to grow on a defined nutrient medium for mammalian cells, i.e., DMEM growth medium as per the experimental studies (Supplementary Data 2). These nutrient-constrained colons and CRC models were then independently integrated with ROS expanded *E. durans* (Supplementary Data 3 and Supplementary Table 1) using the Microbiome Modeling Toolbox^26^ to simulate host–microbe interactions.

**Fig. 6:**
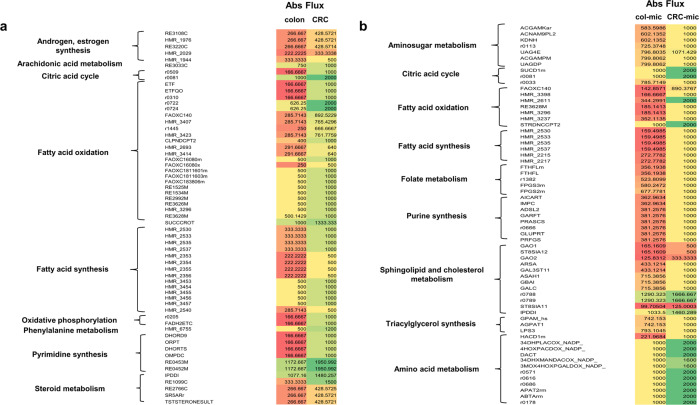
Variation of reaction absolute fluxes highlighting the affected metabolic pathways. **a** Colon vs. CRC and **b** colon-microbe vs. CRC–microbe models.

The first step in investigating the metabolic attributes differentiating healthy colon and CRC was to model the growth of the models, which is a function of the flux obtained through their biomass reactions, respectively using flux balance analysis (FBA). The flux through the biomass reaction of the CRC model was found to be higher (0.0223 mmol/g-DW/h), compared to that of the healthy colon model (0.0003 mmol/g-DW/h), thus, indicating that transformed cells grow and proliferate at relatively higher rates. This defining characteristic of CRC cells is attributed to their ability to cater to the increasing biosynthetic demands for increased proliferation, which was further explored by computing variation in fluxes of the entire metabolic network. To do so, FVA and subsequently, flux distribution ratio (FSr, considering values in the range 0.8 > FSr > 2) analysis of colon and CRC metabolic models, as well as colon-microbe and CRC–microbe models was performed. A surge in biochemical flux was observed through different major metabolic pathways in CRC compared to the colon (Fig. 6a) and CRC-microbe to colon-microbe models (Fig. 6b). Most of these pathways were common to both sets of models and included:

i. Fatty acid oxidation: The reactions participating in fatty acid oxidation catalyzed by Carnitine O-Palmitoyltransferase (CPT; EC:2.3.1.21), Enoyl Coenzyme A Hydratase (ECH, EC:1.3.8.7), and Enoyl Coenzyme A Reductase (EC:1.3.1.38) showed increased fluxes, indicating beta-oxidation of fatty acids was a metabolically pronounced phenomenon in both the CRC and CRC- *E. durans* integrated models.
ii. Fatty acid synthesis: The pathways involving reactions catalyzed by Very-Long-Chain 3-Oxoacyl Coenzyme A Synthase (EC:2.3.1.199) and Very-Long-Chain 3-Oxoacyl Coenzyme A Reductase (EC:1.1.1.330) also had increased flux values, indicative of fulfilling the metabolic requirements of cancer cells.
iii. Amino acid metabolism: Tyrosine metabolic pathway displayed enhanced flux through the reaction catalyzed by 3, 4-Dihydroxymandelaldehyde:NADP + Oxidoreductase (EC:1.2.1.5).
iv. Squalene and cholesterol metabolic pathways were also affected in these metabolic networks. For instance, Isopentenyl-Diphosphate D-Isomerase (IDI, EC:5.3.3.2) which is important in squalene synthesis showed increased flux value in both models.
v. The reaction catalyzed by the enzyme Deoxyuridine Phosphorylase (DURIPP; EC:2.4.2.23) in nucleotide interconversion also showed increased flux.

The relevance of these enzymes and related pathways in cancer cells have been discussed at great length in the Supplementary Discussion. Interestingly, these computational predictions have been concordant with the existing literature findings (summarized in Table 1), thereby supporting the reliability of these models in evaluating metabolic aspects of CRC as a disease. However, certain reactions catalyzed by Prostaglandin I2 synthase (E.C:5.3.99.4; eicosanoid metabolism) and Palmitoyl Coenzyme A Hydrolase (E.C:3.1.2.2; fatty acid oxidation showed reduced fluxes. This contrasts with the findings reported in the literature (Supplementary Discussion).

**Table 1:** Host and host-microbe model predictions in agreement with literature.

Reactions	Comments
CRC specific model predictions	
ATP:Dephospho Coenzyme A 3-Phosphotransferase (EC:2.7.1.24) and Pantothenate 4-Phosphotransferase (EC:2.7.1.33)	These enzymes catalyze the reactions leading to Coenzyme A synthesis, the latter being the acyl carrier participating in various metabolic pathways^60^, and therefore being significant in cancer metabolism. The related enzyme-catalyzed reactions showed increased flux in the CRC model.
Carnitine O-Palmitoyltransferase (EC:2.3.1.21), Enoyl Coenzyme A Hydratase (EC:1.3.8.7), and Enoyl Coenzyme A Reductase (EC:1.3.1.38)	These enzymes catalyze the reactions of fatty acid oxidation metabolic pathways, and their activity is significantly heightened in colon carcinogenesis^61,62^. The related enzyme-catalyzed reactions also showed increased flux values in the CRC metabolic, thus, being consistent with the literature findings.
Isopentenyl-Diphosphate D-Isomerase (EC:5.3.3.2)	This enzyme participates in the mevalonate-isoprenoid biosynthetic (MIB) pathway, which is crucial to CRC metabolism^63^. Interestingly, the CRC metabolic model computational analysis also reported an increased flux through the related enzyme-catalyzed reaction.
Folylpolyglutamate Synthetase (EC:6.3.2.17)	The enzyme is required for the synthesis of folate derivatives (1-C carriers), the excess of which promotes CRC carcinogenesis^64^. The CRC metabolic model showed increased flux value through the related enzyme-catalyzed reactions.

CRC-microbe integrated model predictions	
2-Methylpropanoyl Coenzyme A:Oxygen 2, 3-Oxidoreductase (EC:1.3.99.2) and ‘3-Methylbutanoyl Coenzyme A: (Acceptor) 2, 3-Oxidoreductase (EC:1.3.99.12)	These enzymes catalyze the degradation of BCAAs, the major degradation product being acetyl CoA, which is essentially required for fatty acid synthesis^65^, a metabolic process elevated in CRC cells. The related enzyme-catalyzed reactions in the CRC microbe integrated model also showed increased flux values.
L-Alanine:2-Oxoglutarate Aminotransferase (EC:2.6.1.2)	This enzyme catalyzes the formation of pyruvate and glutamate, the two being important substrates for various major metabolic pathways^66^, and hence relevant to cancer pathogenesis. Computationally, the CRC–microbe integrated model captured the increased activity of this enzyme through increased flux.
17-Beta-Hydroxysteroid Dehydrogenase (EC:1.1.1.35) and Steryl-Sulfatase (EC:3.1.6.2)	These enzymes participate in the steroid biosynthesis (estradiol) metabolic pathway, wherein increased serum levels of estradiol have been reported in colon cancer^67^. The CRC–microbe model also showed increased flux values through the related enzyme-catalyzed reactions.
Arachidonate 5-Lipoxygenase (EC:1.13.11.34)	The enzyme catalyzes the intermediate steps in the synthesis of leukotrienes, and its activity is reported to be elevated in colon cancer cells^68^. The CRC–microbe integrated model captured increased flux for the related enzyme-catalyzed reaction.

### *E. durans*-driven changes in CRC metabolism highlight the relevance of gut microbe

The FSr analysis between the host (CRC and colon)–microbe model sets emphasized changes brought about in the fluxes of the following CRC biochemical reactions in presence of *E. durans* (ROS exposed) model when compared to colon–*E. durans*:

i. Steroid metabolism: In the CRC–*E. durans* integrated metabolic model, steryl sulfatase (STS, EC:3.1.6.2) and Hydroxysteroid (17-Beta) Dehydrogenase 4 (17βHSDs, EC: 1.1.1.35) showed increased flux values.
ii. Oxidative phosphorylation (OXPHOS): One of the crucial highlights of the CRC–microbe integrated metabolic model was increased flux through Sn-Glycerol-3-Phosphate: (Acceptor) 2-Oxidoreductase (EC:1.1.5.3) catalyzed reaction of the OXPHOS pathway. This established the importance of OXPHOS in rewired CRC metabolism when exposed to *E. durans* integration.
iii. Arachidonic acid metabolism: Arachidonate 5-Lipoxygenase (EC:1.13.11.34) catalyzed reaction showed increased flux value in the CRC–microbe model.

Moreover, we also compared fluxes between CRC and CRC–microbe models to identify metabolic changes specifically driven by *E. durans*. These included the following:

i. Phosphatidylinositol (PI) and inositol phosphate metabolism: The flux through various enzyme-catalyzed reactions involved in the PI pathway exhibited increased fluxes in the CRC-microbe integrated model compared to the CRC model alone. These enzymes included 1-Phosphatidylinositol-4-Phosphate 5-Kinase (PIP kinase, EC:2.7.1.68) and Phosphatidylinositol N-acetylglucosaminyltransferase (EC:2.4.1.198).
ii. Glycerophospholipid metabolism: The reaction catalyzing the generation of 1-Palmitoylglycerophosphoinositol and 1-Arachidonoylglycerophosphoinositol showed increased fluxes in the CRC-*E. durans* integrated model. These reactions are catalyzed by phospholipase A2 (EC:3.1.1.4).
iii. Propanoate metabolism: 2-Hydroxybutyrate dehydrogenase (2HBDH; EC:1.1.1.30), catalyzed reaction showed increased flux in the CRC–*E. durans* integrated model.

### Metabolome analysis of the host–microbe models aid in predicting novel therapeutic drug targets in CRC

Another important aspect of our computational studies involving FSr analysis for different sets of metabolic models, viz. CRC and colon; CRC–*E. durans* and colon–*E. durans*, was to identify any affected reactions/enzymes that behaved differentially in CRC (or CRC–*E. durans*) models and could serve as promising new drug targets. These included:

1. Sn-Glycerol-3-Phosphate: (Acceptor) 2-Oxidoreductase (E.C.1.1.3.21) catalyzed reaction of the OXPHOS pathway which displayed increased flux in CRC-microbe integrated model (16.67% increment) indicating that the CRC cells favor oxidative phosphorylation (OXPHOS) over glycolysis.
2. 3, 4-Dihydroxymandelaldehyde Dehydrogenase (E.C.1.2.1.5) catalyzed reactions carried increased flux in CRC and CRC–microbe metabolic models (increase by 62.5%).
3. Catechol O-Methyltransferase (COMT; E.C.2.1.1.6) catalyzed reaction from phenylalanine metabolism in CRC–microbe model showed increased flux by 41.67%.

These novel predictions have been summarized in Table 2 and their significance in CRC has been discussed in detail in the ensuing discussion.

**Table 2:** Host and host–microbe model novel predictions.

Reactions	Comments
CRC specific model predictions	
3, 4-Dihydroxymandelaldehyde:NADP + Oxidoreductase (EC:1.2.1.5)	This enzyme catalyzes the synthesis of DHMA (catechol), a potent anti-oxidant, that inhibits lung cancer^42^. But its role in CRC metabolism/treatment has not been investigated. CRC metabolic model showed increased flux through the related enzyme-catalyzed reaction.

CRC-microbe integrated model predictions	
Sn-Glycerol-3-Phosphate: (Acceptor) 2-Oxidoreductase (EC:1.1.3.21)	This enzyme catalyzes the OXPHOS pathway reaction, which is reported to be rewired in various cancers like prostate and ovarian^39^. The CRC–microbe (when compared with CRC alone) metabolic model captured the switch from glycolysis to the OXPHOS pathway (increased flux through the related enzyme-catalyzed reaction).
Catechol O-Methyltransferase (EC:2.1.1.6)	This enzyme catalyzes the synthesis of S-Adenosyl-homocysteine (SAH) from S-Adenosyl-methionine (SAM) and higher levels of SAH have been implicated in cardiovascular and chronic kidney diseases^45^. The related enzyme-catalyzed reaction showed increased flux in the CRC-microbe model.

**Fig. 7:**
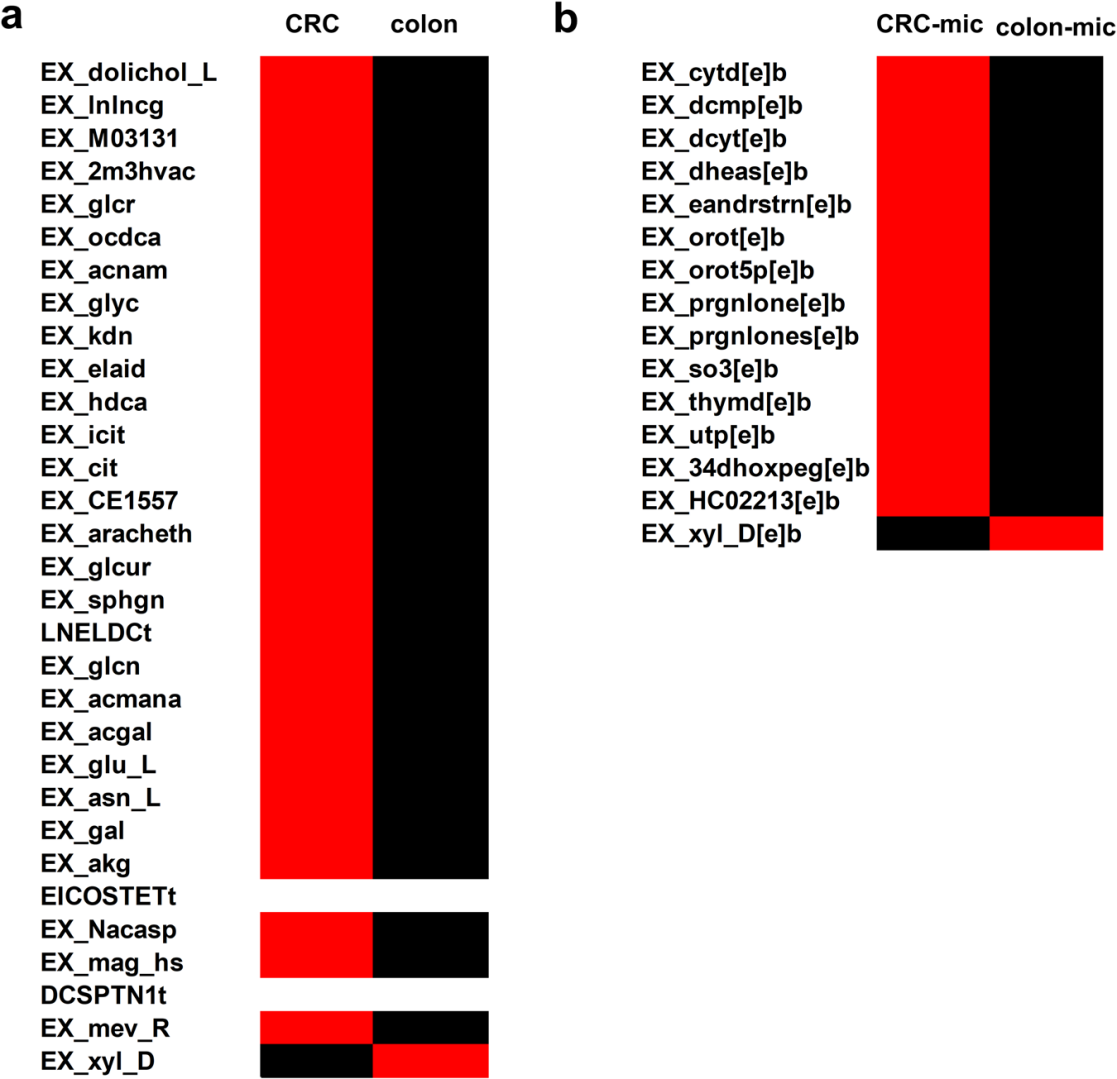
Metabolite secretion profiles of the host and host–microbe models. **a** Colon vs. CRC and **b** colon–microbe vs. CRC–microbe. Exchange reactions with increased fluxes are depicted in red, with the same fluxes in white and reduced fluxes in black.

The secreted metabolite profiles (secretome) of the host and host-microbe models were also analyzed to better understand the metabolic changes associated with CRC metabolism (in the presence and absence of gut microbe). Based upon the FSr analysis of the colon v/s CRC and colon–microbe vs. CRC–microbe models, the exchanges with FSr values falling in the defined range (0.8 > FSr > 2) were further analyzed to establish the secretome of the CRC model. The maximum fluxes for these exchanges were subsequently observed to identify the metabolites with increased secretion in cancer cells (Fig. 7). In case of colon v/s CRC models, the metabolites pertaining to fatty acid metabolism (EX_lnlncg (gamma-linolenic acid), EX_ocdca (stearic acid), EX_hdca (palmitic acid) etc.), sphingolipid and glycolipid metabolism (EX_dolichol_L, EX_acnam (N-acetylneuraminic acid), EX_sphgn (sphinganine)), glucose metabolism (EX_glyc (glycerate), EX_glcn (gluconic acid)), amino acid metabolism (EX_Nacasp (N-acetyl-L-aspartic acid), EX_glu_L, EX_asn_L) showed increased flux in CRC model compared to healthy colon (Fig. 7a). Similarly, a comparison of colon-microbe and CRC–microbe in terms of fluxes through host exchange reactions captured increased fluxes for certain metabolites of importance in cancer (Fig. 7b). These included pyrimidine metabolism (EX_cytd[e]b (cytidine), EX_dcyt[e]b (deoxycytidine), EX_orot[e]b (orotic acid)), steroid metabolism (EX_dheas[e]b (Dehydroepiandrosterone sulfate), EX_eandrstrn[e]b (16a-hydroxydehydroisoandrosterone), EX_prgnlone (pregnenolone)), catechol metabolism (EX_34dhoxpeg[e]b (3,4-dihydroxyphenylglycol)). Further, *E. durans* secreted metabolites can also affect CRC metabolism. In this study, gut bacteria secreted thymidine (intercellular exchange) showed increased flux in CRC–microbe model, thus acting as the point of interaction between the two models. The secretome analysis of cancer cells and its relevance in promoting/controlling the disorder has been discussed in the next section.

## Discussion

The primary aim of this study was to uncover the role of gut microbes in nanoparticle-aided CRC management using guided computational and experimental approaches. Specifically, genome-scale metabolic modeling was utilized to substantiate the metabolic interactions between gut microbe and CRC metabolisms, which were identified through experimental analysis. Firstly, the response of *E. durans* to nanoparticle exposure was examined, as AgNPs have been reported to generate intracellular oxidative stress^27,28^. in biological systems. Despite the notable antioxidant properties displayed by *E. durans*^29^, a lower concentration (25 ppm) of AgNPs resulted in an increased generation of intracellular reactive species (Fig. 3). Furthermore, the hydroxyl levels increased noticeably during the late log phase in nanoparticle-treated cultures, indicating that AgNPs are potent inducers of ROS. In turn, the nanoparticle-generated oxidative stress has been reported to affect the cellular metabolism in different biological systems, thereby increasing the productivity of certain metabolites (for instance, xanthan gum and pyocyanin in *Xanthomonas campestris*)^30,31^. Based on these findings, it was hypothesized that increased ROS levels may impact the gut microbial metabolism and secretion profiles of metabolites, such as folate, while the latter being of major relevance in key metabolic pathways.

To test this hypothesis, we used the systems biology modeling approach (COBRA toolbox)^32^ to analyze the systems-level effects of ROS on the metabolism of *E. durans*. The modeling framework assisted in establishing the critical link between folic acid metabolism and other important intermediary metabolic pathways (i.e., amino acid, energy, lipids, as well as SCFA metabolism) in the gut microbe exposed to ROS (Supplementary Table 2). For instance, the minNorm analysis indicated that the addition of ROS to the model system affected glycine hydroxymethyl transferase (EC:2.1.2.1)^33^ catalyzed reaction, wherein the 3-carbon serine serves as one of the major sources in transferring one-carbon moiety to tetrahydrofolate (THF) to form 5, 10-methylene THF (MLTHF). A positive flux was generated in this reaction, implying an increased release of folate derivatives, thereby affecting folate metabolism. Similarly, an increased flux was observed in methylenetetrahydrofolate dehydrogenase (EC:1.5.1.5) catalyzed reaction that synthesizes METHF (methenyl THF) from MLTHF (derived from serine metabolism), thus associating energy (NADPH) and nucleic acid metabolism with folate metabolism^34^.

Interestingly, the novel link between folic acid derivatives and SCFA (propionate) metabolism was predicted by the model (Fig. 4). The two enzymes linking these essential pathways were malonyl CoA pyruvate carboxytransferase (EC:2.1.3.1) and 2-oxobutanoate formate lyase. Both these enzymes are required for synthesizing propionate CoA, which further participates in propionate metabolism. The role of ROS in increasing the folate levels was further validated through experiments, wherein an increase in extracellular folate levels was observed in AgNP treated bacterial culture (Fig. 5a). Thus, this is the first study of its kind that establishes the interplay between gut bacterial metabolism and AgNP-generated intracellular oxidative stress.

Moreover, the interactions between *E. durans* and CRC were extensively modeled to assess the role of gut microbes in disease etiology. To do so, multi-omics (proteomics and transcriptomics) data-driven metabolic models of CRC and colon were generated (refer methods section). These models were used to analyze distinct metabolic features of CRC cells often represented through rapid growth and division in tumor cells when compared to their healthy counterparts (colonocytes)^35^. A higher rate of cell proliferation was observed in the CRC model in terms of increased biomass flux (i.e., approximately 98%), as compared to the healthy colon model, thus corroborating the existing literature reports^36^. Moreover, these tissue-specific models were also able to capture various metabolic pathways (Fig. 6a), responsible for providing various biosynthetic components (ATP, NADPH, NAD^+^, acetyl-CoA, and amino acids) to the transformed cell for sustaining its growth and proliferation.

Further, to understand the effect of gut microbes on CRC metabolism, the host–microbe interactions were simulated by constraining the host-microbe integrated model as per the experimental conditions. These metabolic interactions can aid in deciphering the interplay between gut microbes and tumor cells, and hence be advantageous in devising novel therapeutic targets in the context of AgNP-based CRC treatment. The metabolites secreted by gut microbes also affected CRC cell viability, as a significant reduction in viability was observed in HCT 116 cells treated with supernatants obtained at the 9th h of bacterial culture growth—both control and AgNP treated cultures (Fig. 5b). Moreover, the extracellular folate concentrations measured in AgNP treated cultures at the 9th h of bacterial growth were equivalent to the optimal-cytotoxic concentration of synthetic folic acid that resulted in increased cell death (compared to control). These experimental findings observations found that oxidative stress heightened the production of folate to optimal-cytotoxic levels, affecting cancer cell viability.

Contrary to experimental findings, the CRC–microbe model was unable to capture the anti-cancer effects of gut bacterial metabolite(s), as no decrease in flux was observed in the flux through the CRC-biomass reaction. Typically, an oncogenic transformation operates at multiple levels of signaling, metabolic, and regulatory variations, all culminating in the re-wiring of cellular metabolic pathways^12,37^. However, owing to the limitations of GSMs, these models were unable to capture other significant cellular aspects (i.e., signaling and regulatory) associated with metabolism. Nonetheless, the tissue-specific models (CRC and CRC–*E. durans*) effectively captured various metabolic pathways that contribute to aberrant CRC metabolism (Fig. 6b).

One of the major outcomes of this study was the agreement observed between the computational predictions and literature findings for the affected pathways in CRC and CRC–microbe models. A detailed review of the same has been discussed in the Supplementary Information (Supplementary Figs. 4–6) along with additional analysis such as computing shadow prices, flux enrichment analysis, etc. (details in Supplementary Discussion). Furthermore, these metabolic models were able to predict enzymatic reactions that could serve as novel drug targets while managing CRC (Table 2). For instance, the constraint-based CRC-microbe integrated metabolic model showed increased flux through Sn-Glycerol-3-Phosphate: (Acceptor) 2-Oxidoreductase (EC:1.1.3.21) catalyzed reaction of the OXPHOS pathway when compared with colon–microbe model. Experimental studies have shown that the cancer cells derive their energy mainly through aerobic glycolysis (Warburg effect)^38^. In such cases, the cells prefer to metabolize carbohydrates through glycolysis, despite having completely functional mitochondria, thus not resorting to a more efficient oxidative phosphorylation (OXPHOS) pathway. However, recent studies have shown that certain cancers (prostate and ovarian) exhibited a switch from glycolysis to OXPHOS for energy generation^39^. This change in energy metabolism is another prominent characteristic feature of cancer metabolism, which makes the OXPHOS pathway an effective target for cancer therapeutics. This phenomenon has not been reported in CRC-related experimental studies this far. In addition to linking carbohydrate and lipid metabolism, this enzyme also catalyzes the generation of hydrogen peroxide (H_2_O_2_), the latter resulting in increased intracellular oxidative stress^40^. This chronic oxidative stress in turn is conducive to the pro-inflammatory micro-environment of CRC tissues. Thus, given its significance in the OXPHOS pathway, this enzyme can serve as a novel chemotherapeutic drug target.

Another enzyme, 3, 4-dihydroxymandelaldehyde: NADP + oxidoreductase (EC:1.2.1.5) facilitates the conversion of 3,4-dihydroxymandelaldehyde to 3,4-dihydroxymandelic acid (DHMA) in tyrosine metabolism. DHMA, a catechol, is a norepinephrine metabolite known to possess strong antioxidative potential^41^. Certain in vitro studies have shown that catechol inhibits the growth of lung cancer, and therefore possesses anticancer attributes^42^. However, its role in CRC metabolism and treatment has not been investigated. Interestingly, 3, 4-dihydroxymandelaldehyde: NADP + oxidoreductase catalyzed reactions showed increased flux in CRC and CRC–microbe metabolic models, thus, favoring its importance as a therapeutic target while designing novel cancer treatment drugs.

Catechol O-methyltransferase (COMT; EC:2.1.1.6) catalyzes the synthesis of S-Adenosyl-homocysteine (SAH) from S-Adenosyl-methionine (SAM), the latter being a major methyl donor in transmethylation reactions that can downregulate the expression of oncogenic promoters by reversing DNA hypomethylation, thereby inhibiting tumor growth^43^. Furthermore, this tumor-inhibiting attribute of SAM is well established in gastric and colon cancers^44^. In CRC–microbe model, an increase in flux through this reaction was observed (41.67%). On the other hand, high SAH serum levels inhibit SAM-dependent methyltransferases (that catalyzes methionine to SAM conversion). Elevated serum concentrations of SAH have been reported in chronic diseases like cardiovascular and chronic kidney diseases^45^. However, the role of SAH has not been established in colon cancer yet. The reduced levels of SAM (on account of increased flux through COMT catalyzed reaction in the CRC–microbe model) can result in increased cancer cell proliferation and growth making COMT a promising drug target, an aspect that can motivate future experimental studies.

To better understand the anomalous behavior of CRC, the secretome profiles obtained from medium constrained models focused on the metabolites accountable for deviant cellular characteristics observed in CRC by altering the tumor micro-environment. The models were constrained as per the DMEM composition, wherein various macro- and micro-nutrients were provided as substrate inputs. Several metabolite secretions were affected in diseased conditions compared to a healthy colon. For instance, increased secretion of alpha-ketoglutarate (EX_akg), an intermediate of the tricarboxylic acid cycle, reinstated its relevance in the oxidation of fatty acids, amino acids, and glucose^46^. Other metabolites partaking in fatty acid metabolism, glycolysis, sphingolipid, and amino acid metabolism also showed increased exchange fluxes in the CRC model, compared to the colon (Fig. 7a). An essential metabolite, N-acetyl-l-aspartic acid (EX_Nacasp) which has a prominent role in promoting tumor growth and can act as a potential target for anticancer therapy^47^ also showed increased secretion in the CRC model.

Similarly, CRC-microbe secretome analysis also featured elevated fluxes in various metabolites secretions (Fig. 7b) like orotic acid (EX_orot[e]b) and its derivative (EX_orot5p[e]b), which is a powerful tumor promoter in hepatocellular carcinoma^48^. Pregnenolone (EX_prgnlone[e]b) and sulfites (EX_so3[e]b) which promote prostate cancer^49^ and ulcerative colitis, potentially leading to colon cancer^50^ respectively, also showed increased secretion in the integrated model. In addition to the tumor-promoting metabolite secretions, dehydroepiandrosterone sulfate (EX_dheas[e]b) a key adrenal steroid that is known to have some protective role in CRC^51^ also showed elevated levels in the host–microbe integrated model. Moreover, it was thymidine secreted by *E. durans* that acted as an important metabolite connecting the microbe metabolism with that of the host. Increased extracellular transport of thymidine from microbe to CRC could have possibly boosted the latter’s pyrimidine metabolism compared to the healthy colon, as captured and supported by the FSr analysis. Thus, all the computational predictions cumulatively emphasized the applicability of GSMMs in comprehending CRC metabolism and potential drug targets.

To summarize, CBM effectively predicted the association between ROS and folate metabolism, which was also verified through experiments. Moreover, context-specific models of CRC and colon could accurately capture the fundamental biochemical differences between a healthy colon- and CRC-cell, further demonstrating the metabolic adaptations essential for CRC survival. The integration of CRC and gut microbe metabolic models to simulate host-microbe interactions was beneficial in understanding CRC metabolism in presence of a gut microbe. These metabolic interactions can help mimic the interplay between gut microbes and tumor cells under in vivo conditions and can be advantageous while devising novel therapeutic targets in the context of AgNP-based CRC treatment.

## Methods

### Characterization of silver nanoparticles (AgNPs)

Silver nanoparticles (AgNPs) were obtained from Sigma Aldrich (catalog No. 7440-22-4). Size distribution analysis was carried out using dynamic light scattering and zeta potential was measured using Horiba Scientific Nanopartica nanoparticle analyzer (SZ-100). A scanning electron microscope was used to study NP–bacteria interaction.

### Bacterial culture and treatment with AgNPs

*E. durans*, a facultative aerobe, procured from MTCC (MTCC No. 3031) was used as the model organism. The bacterial culture was grown in shake flasks containing MRS broth at 37 °C, 180 rpm, in a shaker (Scigenics Orbitek). The total cell concentrations at different time points were measured through optical density (cell scatter) at 600 nm (JASCO V-630 Spectrophotometer), and a comparison with a standard plot of OD vs. cell concentration was done.

The AgNPs (25–250 ppm) were dispersed in the medium using a water bath sonicator. The medium was then inoculated with the appropriate volume of the subculture (inoculum), such that the OD value at the 0th h was 0.1.

### Quantifying intracellular ROS concentrations

Intracellular ROS were measured by following the procedures from literature^52^. 3′-(p-amino-phenyl) fluorescein, APF (Invitrogen, USA) and dihydroethidium, DHE (Sigma-Aldrich, India) were used to detect hydroxyl and superoxide radicals, respectively. The samples (microbial cell cultures at different time points) were incubated with the dyes (2 mM DHE at 37 °C and 5 mM APF at room temperature) for 30 min. Upon detection through a fluorescent spectrophotometer, the hydroxyl and superoxide radical concentrations were evaluated using the standards plots of hydrogen peroxide and potassium superoxide, respectively. The concentration of superoxide and hydroxyl radicals was reported in nmol/(g-cell).

### Sample preparation for folate estimation and HPLC analysis

The bacterial culture was harvested every 3 h and culture volume corresponding to 10 OD was used for sample extraction. The extracted culture volume was centrifuged, and the bacterial pellet obtained was suspended in 1 ml of milli Q water. It was then sonicated (Q Sonica sonicator), at an amplitude of 70%, for a processing time of 4 min (pulse on and off time being 2 s). The sonicated sample was then placed in a water bath at 100 °C and subjected to heat for 5 min to release any folate bound to the folate binding proteins. The cell-free extract was obtained by centrifuging the sample. The supernatant was collected, filtered, and used further for folate estimation. For quantifying extracellular folate, released by the bacterial cells into the growth medium, 1 ml of culture was collected every 3 h and the filtered supernatant was used for HPLC using UFLC Shimadzu HPLC setup. C18 Hypersil column (25 cm*4.6 mm, 5-micron spherical packing)^53^. Carbinol in 0.05 M KH_2_PO_4_ which was used as the mobile phase was filtered through 0.46-micron filters before use and then sonicated in a bath sonicator for 10 min for degassing the solution. The flow rate was maintained at 0.4 mL/min.

Folic acid (Himedia, catalog No. CMS175) was used as standard (0–125 μM) in the estimation of folate in samples (cell extracts and bacterial supernatant).

### Treatment of HCT 116 with bacterial supernatant

HCT 116 (colon cancer cell line) was obtained from Dr. Bert Vogelstein, John Hopkins University, Baltimore, USA. The tumor cells were grown in Dulbecco’s Modified Eagle Media (DMEM), with 5% serum. The cells were seeded and grown in a 96-wells plate and were treated with bacterial supernatant (control and 25 ppm AgNPs), and different concentrations of synthetic folic acid. MTT cell proliferation assay was then performed to quantify cell viability on exposure to the drug after 48 h of treatment^54^.

### Modeling gut bacteria—ROS interplay: constraint-based model formulation

In our study, the unconstrained *E. durans* GSMM was downloaded from the VMH database (https://vmh.uni.lu/) and expanded using ROS reactions curated from the literature.

rBioNet was used to add the different reactions (ROS reactions; missing transport and exchange reactions; sink and demand reactions) to the model. rBioNet enables the user to add these reactions in a quality-controlled manner, by exempting sources of manual error^55^. A total of eight ROS reactions and 11 metabolites were added to the model. These ROS reactions focused on the biochemical interactions between reactive species (predominantly superoxide, hydroxyl radical, and nitric oxide), and amino acids^56^, and nucleic acids^57^. The resulting reconstruction (with ROS reactions) was then merged with the downloaded metabolic model, thus, formulating the ROS expanded model (Supplementary Data 1). FBA was carried out on the newly added reactions individually to check if the reactions were blocked (carrying zero net flux). Blocked reactions might result from incomplete reaction information about the consumption of substrates or the generation of products. These blocked reactions were then resolved by adding complete metabolic reactions and pathways from the literature). In case there was no literature evidence supporting reactions pertaining to the blocked reaction, the reaction was un-blocked (by adding demand or sink reactions for products and reactants, respectively). For *E. durans* model, seven demand reactions and one sink reaction were added. Upon un-blocking the newly added reactions, dead-end metabolites were then identified. Dead-end metabolites are the ones that are either produced or consumed but never both. These dead-end metabolites were also resolved using literature information for adding the required reactions to the model. The debugged model was medium constrained as per MRS medium composition.

FVA was then performed that provided the flux values (minimum and maximum) FSr, which is the ratio between absolute flux values of reactions for healthy model (original *E. durans*) to absolute flux values of reactions for disease model (ROS-expanded *E. durans*) was calculated. The range for FSr is user-defined. The reactions with FSr values in the range 0.8 > FSr > 2 were identified. These were the reactions that were considered as affected due to the addition of ROS reactions to the model. minNorm analysis was then carried out on these reactions. minNorm function in MATLAB is a tool for frequency estimation of the data vector, for a given function.

### Context-specific model building

Recon 3D, a human metabolic reconstruction, consisting of metabolic reactions and their corresponding enzymes/genes was used to generate the colon and CRC condition-specific tissue models. The ‘omics’ data (obtained from HPA)^23^ for healthy and CRC colon was mapped onto Recon 3D, and the resultant model was derived using iMAT algorithm^25^ to provide a better understanding of the molecular phenomena occurring in cancer and normal cell types. iMAT is an integrative metabolic analysis tool, that allows the integration of transcriptomic and proteomic data with genome-scale metabolic networks to calculate fluxes associated with enzyme metabolized reactions. It works best for capturing reactions/pathways associated with the given data input. This is advantageous in cases where a single objective is not obeyed/maintained, for example, mammalian cells. The reactions are grouped in three categories based on the expression levels of proteins as high, medium, and low (as per HPA). A data matrix is constructed, wherein the primary reaction set represents reactions with a high and medium confidence level to be present in the cell type, and this data matrix is fed in the iMAT algorithm. The gap-filling was then done using MILP (mixed-integer linear programming) approach, maintaining the defined stoichiometric and thermodynamic constraints. The preliminary models once generated are further curated to satisfy the sanity checks as per COBRA toolbox standards. Further, to the healthy colon model, transport reactions were added as reported in literature^58^, and the biomass reaction was modified to exclude replicating precursors and include dependency on cellular folate^59^. However, the CRC model was retained with the biomass reaction as in Recon 3D. Additionally, both the healthy and CRC colon models were expanded to include exchange reactions for all the metabolites appearing in the extracellular compartment.

### Microbiome modeling toolbox

The Microbiome Modeling Toolbox^26^ provided by the COBRA toolbox was used to integrate host and microbe models. This toolbox is primarily used for modeling microbe-microbe/host-microbe interactions, as well as personalized microbial communities.

### Statistical analysis

All cultures and measurements were carried out in triplicates (each subjected to at least three technical replicates). Values have been reported as mean ± SD (please refer to individual results in the Results section). One-way ANOVA (level of significance, *α* = 0.05) and Tuckey’s multiple comparison tests were carried out.

### Reporting summary

Further information on research design is available in the Nature Research Reporting Summary linked to this article.

## Supplementary information

Supplementary Information

Supplementary Data 1

Supplementary Data 2

Supplementary Data 3

Reporting Summary

## Data Availability

The AGORA resource is freely available at the Virtual Metabolic Human website (https://vmh.life). The models used in the study are growth medium constrained and have been provided as excel sheets in Supplementary Data 1–3.

## Code availability

MATLAB codes for performing simulations and data analyses can be accessed at https://opencobra.github.io/cobratoolbox/latest/modules/analysis/index.html and Supplementary Note.
