# Supplementary material for: Metabolic modeling of host–microbe interactions for therapeutics in colorectal cancer
Source: NPJ Syst Biol Appl. 2022 Jan 19;8:1. doi: 10.1038/s41540-021-00210-9 (PMC8770697; doi:10.1038/s41540-021-00210-9)
Supplement: Supplementary file 1 — Supplementary Information [file 41540_2021_210_MOESM1_ESM.pdf]

Supplementary Information

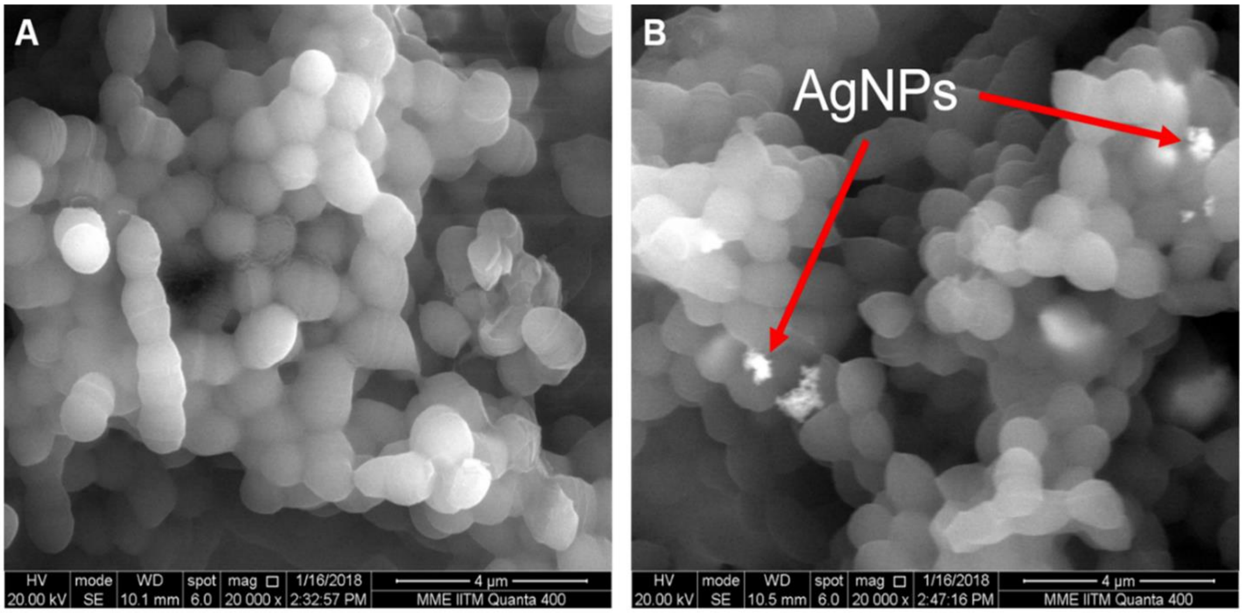

Supplementary Figure 1: Scanning electron microscopy showing A) bacteria alone, B) AgNPs interacting with the bacterial cells.

The silver nanoparticles used in this study were first characterized for their size and stability in the bacterial culture medium. The stability of the nanoparticles was assessed through zeta potential measurement. A negative value (-13 ± 0.95 mV) indicated better stability of the dispersed nanoparticles. The higher stability also indicated that the well dispersed nanoparticles could effectively interact with the bacterial cells in the culture and consequently affect their viability when used at different concentrations.

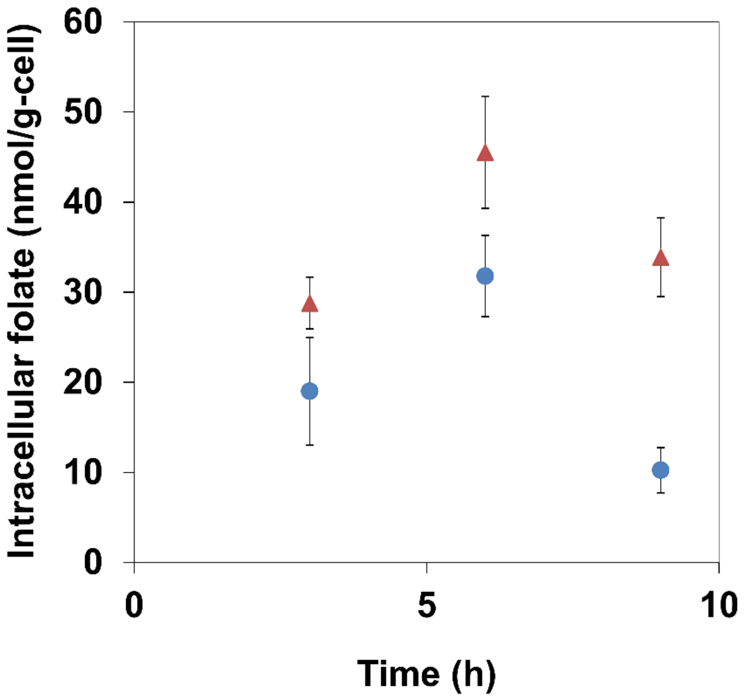

Supplementary Figure 2: Intracellular folate-time profile in the absence and presence of AgNP generated oxidative stress (● indicates control culture; ▲ indicates culture treated with 25 ppm AgNP). Values are expressed as mean ± S.D., n = 3.

HPLC analysis of intracellular folate levels in AgNP-treated bacterial culture showed a maximum folate concentration at the 6th hour of microbial growth. The specific intracellular folic acid concentration was 45.53 ± 0.012 nmol/g-cell in AgNP-treated culture, which was 49% higher compared to control.

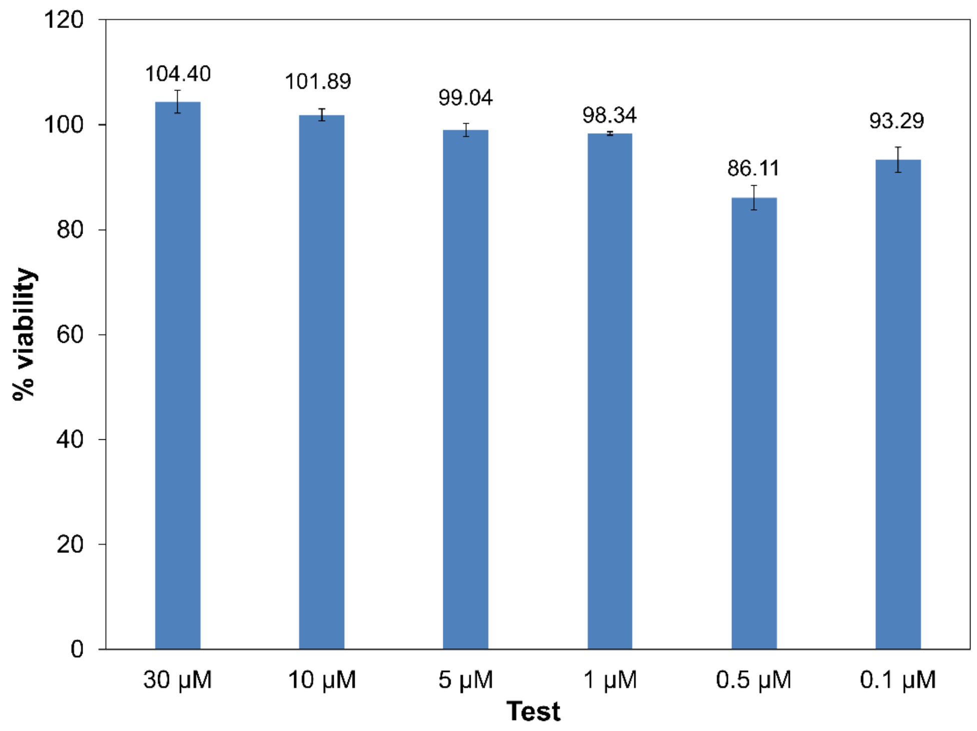

Supplementary Figure 3: Variation in viability of HCT 116 cells upon exposure to different concentrations of folic acid. Values are expressed as mean ± S.D., n = 3.

As expected, higher concentrations of folic acid (5-30 µM) resulted in increased viability of cancer cells. On the contrary, unexpectedly, lower concentrations (1-0.1 µM) reduced the cell viability. The optimal-cytotoxic concentration was around 0.5 µM folic acid, which resulted in decrease of viability by 13.89% when compared with the appropriate control. The reduction in cell viability at lower concentrations of folic acid was statistically significant and exhibited Hormesis effect [1]. Hence, this biphasic dose response phenomenon or the Hormesis effect, exhibited by folic acid needs to be studied further to establish its modality as an anti-cancer drug.

Supplementary Table 1: Statistics of medium constrained genome-scale metabolic models

Model contents	Statistics				
	Microbe	ROS microbe	Colon	CRC	Healthy Colon-microbe (CRC-microbe)
Total reactions	1279	1313	7832	8439	13589 (14698)
Metabolic reactions	871	879	4908	5030	5787 (5909)
Transport reactions	158	167	1891	2117	4201 (4531)
EX_+ DM_+ sink_	250	267	1033	1292	3601 (4258)
Total metabolites	1031	1049	4627	5008	7861 (8627)

Supplementary Table 2: *E. durans* model predictions

Findings in agreement with literature	
Reaction Name	Comments
Glycine hydroxymethyltransferase, reversible (E.C.2.1.2.1)	Glycine, serine, and threonine metabolism was affected upon addition of reactive species to the *E. durans* genome-scale metabolic network, thereby affecting the cellular folate pool.
Methylenetetrahydrofolate dehydrogenase (NADP) (E.C.1.5.1.5)	This enzyme-catalyzed reaction generated positive flux in ROS expanded *E. durans* metabolic model. Moreover, this enzyme links folate metabolism with NADPH and serine metabolic pathways.
Novel findings	
Malonyl CoA pyruvate carboxytransferase (E.C.2.1.3.1) and 2-Oxobutanoate formate lyase	The association between SCFAs and folic acid metabolism was captured for the first time through flux analysis of the ROS expanded microbe model.

Supplementary Discussion

The colon-microbe and CRC-microbe integrated models consisted of 13589 and 14698 reactions, respectively. As per the modeling analysis, there were a total of 104 bacteria secreted metabolites that could be taken up by the host (CRC) cell, of which, 56 metabolites were predominantly required for increasing flux through CRC biomass reaction. It was found that any increment in the biomass flux was directly proportional to the uptake fluxes (mmol/g-DW/h) of these metabolites. These significant metabolites included 20 amino acids, vitamins, and other micro-nutrients, which were the participating species in the biomass reaction, and therefore, can explain the increased biomass flux values. However, the other 48 metabolites showed no quantitative effects on the net biomass flux. The specific interactions among various microbe secreted metabolites, as well as their cumulative effects on the flux through biomass reaction were not considered in our modeling studies, which could possibly justify the reason why no significant quantitative change was observed in the biomass flux.

To understand the metabolic differences between models, flux span ratios were calculated, where FSr is defined as the difference between maximum and minimum flux.

$$FSr = abs(minFlux - maxFlux)_{rxn\_A(1)} / abs(minFlux - maxFlux)_{rxn\_A(2)} \quad (1)$$

Here, *rxn*_A(1) represents reaction named A in model 1 (colon or colon-microbe) and *rxn*_A(2) represents reaction named A in model 2 (CRC or CRC-microbe).

Based on the FSr values (0.8>FSr>2), the following pathways were found to be affected:

1. Purine metabolism:

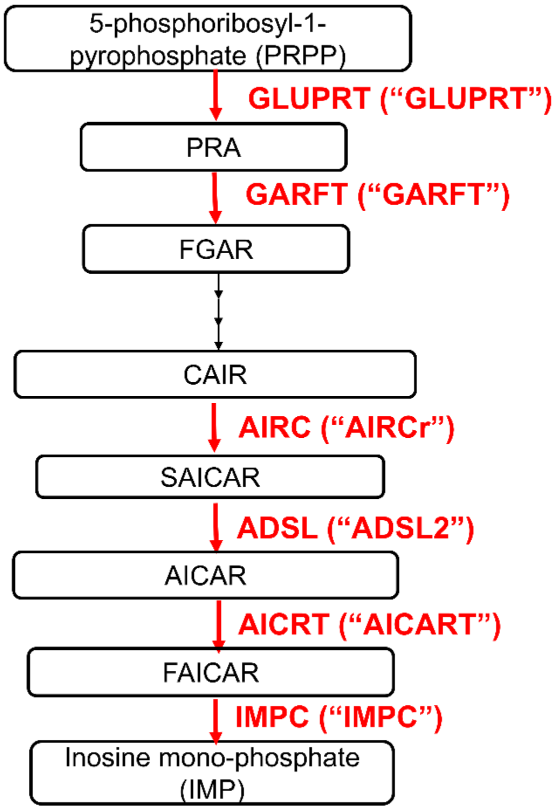

Supplementary Figure 4: Altered purine metabolism in CRC cells, compared to the healthy colon model, wherein, the enzyme catalyzed reactions (highlighted in red), showed increased fluxes in the CRC model.

The CRC model highlighted increased flux through various enzymes participating in purine synthesis and catabolism. Reactions catalyzed by the enzymes like Purine-Nucleoside Phosphorylase (Guanosine)-PUNP, Glutamine Phosphoribosyldiphosphate Amidotransferase (GLUPRT), Phosphoribosylglycinamide Formyltransferase (AIRC), Phosphoribosylglycinamide Formyltransferase (GARFT), Adenylosucccinate Lyase (ADSL), Phosphoribosylaminoimidazolecarboxamide Formyltransferase (AICRT) and IMP Cyclohydrolase (IMPC) carried increased flux in the CRC metabolic model, compared to the healthy colon model. In the de novo purine synthesis pathway, PUNP phosphorylates guanosine to synthesize guanine, and GLUPRT, AIRC, GARFT, ADSL and IMPC catalyze further steps [2].

2. Pyrimidine metabolism:

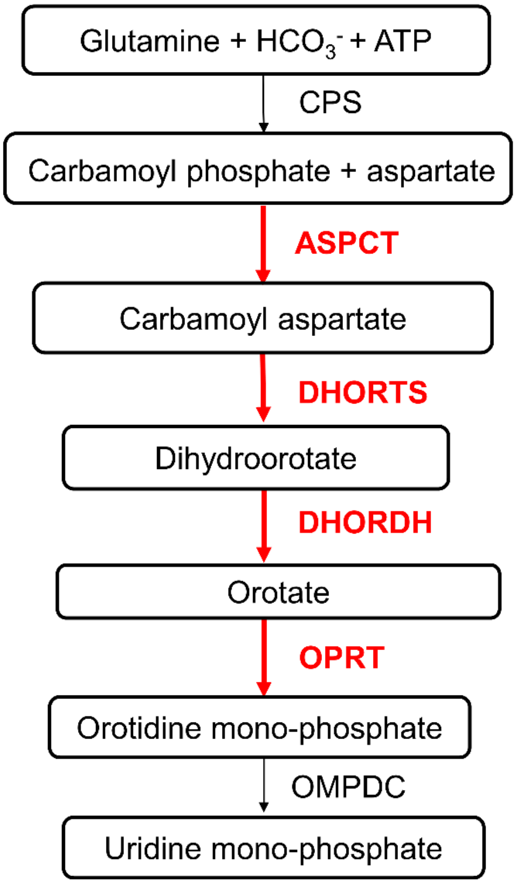

Supplementary Figure 5: Altered de novo pyrimidine synthesis pathways in CRC-microbe integrated model, wherein, the enzyme catalyzed reactions (highlighted in red), showed increased fluxes in the CRC-microbe integrated model.

Certain important reactions catalyzed by dihydroorotate dehydrogenase (DHORDH,'DHORD9'), aspartate carbamoyltransferase (ASPCT, 'ASPCTr'), orotate phosphoribosyltransferase ('ORPT') and dihydroorotase ('DHORTS') showed increased flux in host-microbe model. ASPCT is known to catalyze the second step of de novo pyrimidine synthesis, i.e., formation the carbamoyl aspartate from aspartate and carbamoyl phosphate. Modeling analysis showed increased activity of this enzyme (implying increased production of carbamoyl phosphate) in the CRC tissue samples. The subsequent reactions in the de novo pyrimidine synthesis pathway also showed increased flux. Both ASPCT and DHORTS are part of the CAD protein, and the gene coding the latter is highly

expressed in many tumors. DHORDH is required for the formation of the pyrimidine ring [2]. It is the only enzyme that can catalyze the conversion of dihydroorotate to orotate, therefore, it is of utmost importance for synthesizing uridine monophosphate (UMP) and the inhibition of this enzyme causes suppression of the de novo pathway.

Moreover, the DHORDH catalyzed reaction is coupled ubiquinone reduction to generate ubiquinol [3] and is linked to the electron transport chain (ETC). This finding has been supported in the *in-silico* results as well, where ubiquinone synthesis reaction showed increased flux. Interestingly, multiple types of cancers, such as AML, have increased DHODH activity [4]. But the same has not been reported in CRC samples yet. The CRC-microbe model also showed an increased flux through deoxy-uridine phosphorylase (DURIPP), required to catalyze uracil synthesis. Elevated levels of uracil and thymidine are toxic to the cells, causing replication associated errors.

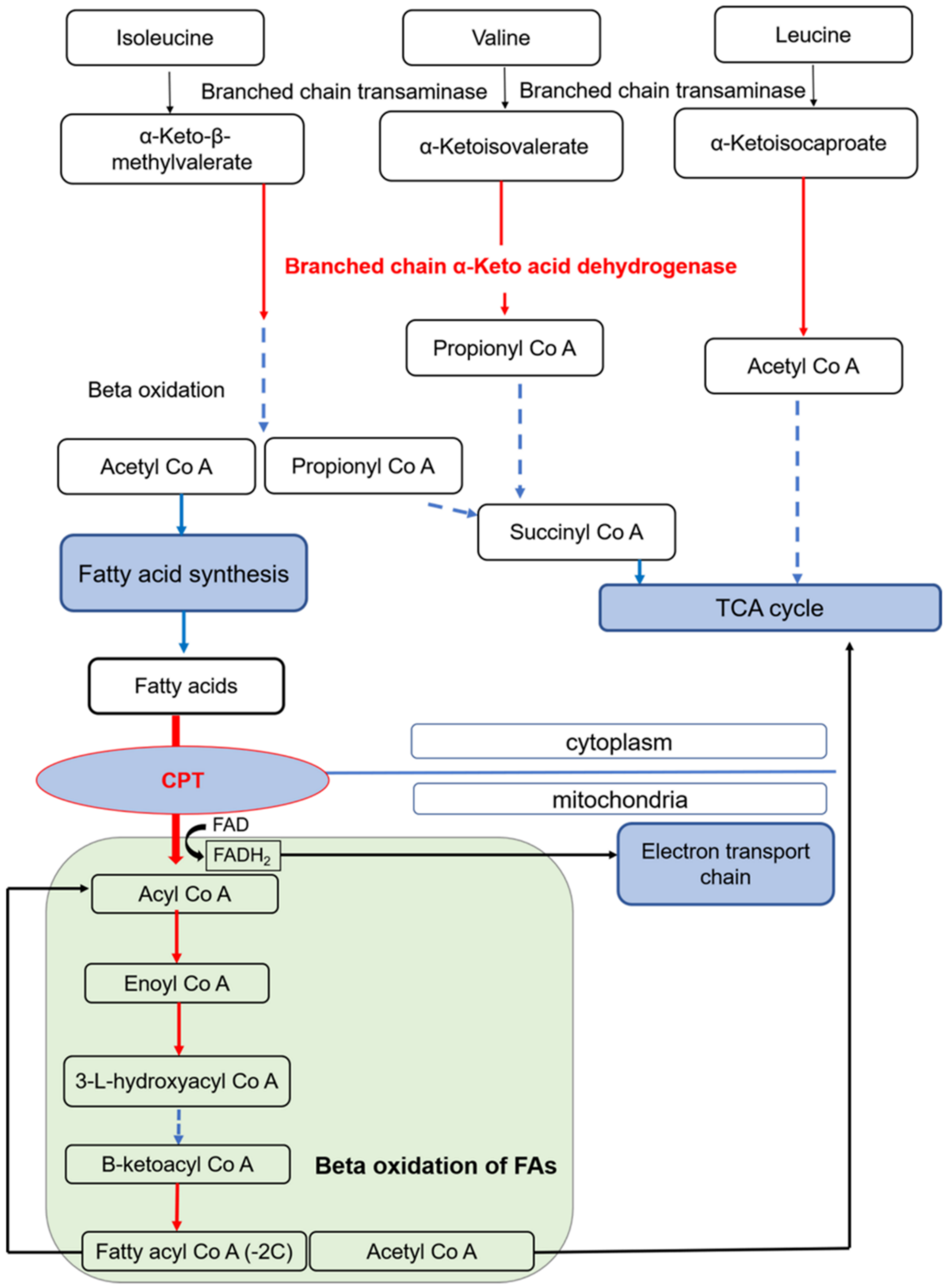

Supplementary Figure 6: Association between the major metabolic pathways showing heightened flux activity (arrows in red) in the CRC-microbe integrated model.

3. Fatty acid metabolism:

Cancer pathogenesis involves increased expression of genes encoding monoacylglycerol lipase, an enzyme of lipid metabolic pathway that releases free FA from lipid stores [5]. The rate controlling

step in FAO is the import of fatty acid into the mitochondria catalyzed by tissue-specific isoforms of Carnitine O-Palmitoyltransferase (CPT). Enhanced activity of CPT increases FAO and ATP production and protects cells from glucose deprivation or hypoxia induced cytotoxicity [6]. Acyl-CoA dehydrogenases (ACADs) catalyzes the first and rate-determining step of peroxisomal beta-oxidation of fatty acids [7], whereas ECH catalyzes the second step of the mitochondrial FAO. Enoyl Coenzyme A Hydratase (ECH) metabolizes fatty acids to generate acetyl CoA and ATP by hydrolyzing the double bond between the second and third carbons on 2-trans/cis-enoyl-CoA [8]. The role of ECHS1 has been implicated in breast, prostate, colon, and liver cancer, as per the literature [9]. In addition, the role of ECH has been identified in signal transduction, where it acts as a novel interacting protein of signal transducer and activator of transcription 3 (STAT3) [10]. Acetyl CoA acyltransferase catalyzes the final step of FAO, wherein, acetyl CoA is released and the CoA ester of a fatty acid two carbons shorter is formed [11]. The acetyl CoA is then consumed as substrate in the energy pathways. Upon analysis of the CRC-microbe and CRC models, these enzyme catalyzed reactions associated with fatty acid oxidation showed increased fluxes, thus capturing the significance of fatty acid oxidation in cancer in these metabolic models.

4. Amino acid metabolism:

Valine (Val), leucine (Leu) and isoleucine (Ile) are the branched-chain aliphatic amino acids (BCAAs), and their degradation products include acetyl Co A, which is an important substrate for fatty acid synthesis [12]. Besides their role as respiratory substrates, these amino acids also play a structural and signaling role [13]. The CRC-microbe integrated metabolic model showed an increase in fluxes for various enzyme catalyzed reactions associated with BCAAs degradation, thereby supporting increased BCAAs catabolism in cancer cell.

5. Energy metabolism:

L-Alanine:2-Oxoglutarate Aminotransferase (also known as Alanine aminotransferase, ALT) is an important enzyme, which catalyzes the formation of pyruvate and glutamate by transferring an amino group from alanine to alpha-ketoglutarate in alanine cycle [14]. Pyruvate is a critical metabolite, which participates in variety of metabolic pathways like glucose, amino acids, and lipid metabolism [15]. Glutamate serves as a precursor metabolite in glutamine formation. Glutamine again being a key metabolite, is a 1-carbon (1-C) donor in nucleic acid and amino acid synthesis. The increased activity of ALT, in terms of metabolic flux was captured in CRC-microbe model.

6. Steroid metabolism:

Recent studies have emphasized the importance of sex hormones (specifically estrogen) in breast cancer pathogenesis [16]. Increased serum levels of estradiol (E2), the active form of estrogen, has been reported in colon cancer [17]. In the CRC-microbe model, steroid sulfatase (STS) and Hydroxysteroid (17-Beta) Dehydrogenase 4 (17βHSDs) showed increased flux values. STS catalyzes the hydrolysis of steroid sulfates (estrone sulfate, ES1) to their unconjugated, biologically active forms [18]. 17β-HSD1 then reduces the estrogen (E1) obtained through the activity of STS into estradiol [19]. The genes coding both these enzymes showed overexpression in many breast tumors [20].

7. Squalene synthesis:

Mevalonate pathway, which is responsible for isoprenoid and cholesterol synthesis, is of major significance in cancer metabolism [21]. The intermittent metabolic reactions in the pathway have often been targeted in cancer therapeutics [22]. For instance, isopentenyl diphosphate isomerase (IDI, 'IPDDI') is one of the important enzymes catalyzing the conversion of isopentenyl pyrophosphate (IPP) to dimethylallyl pyrophosphate (DMAPP) in the mevalonate-isoprenoid biosynthetic (MIB) pathway. The metabolic models (both CRC and CRC-microbe) used in our study also highlighted

the increased activity of this enzyme, which is in consensus with the literature findings. Moreover, previous experimental studies in human prostate cancer and CRC have reported that IDI can serve as an important drug target to induce cell death signaling [23,24].

8. Hexosamine biosynthetic pathway (HBP):

Hexosamine Biosynthetic Pathway (HBP) operates to produce UDP-N-Acetylglucosamine (UDP-GlcNAc), an important substrate required in protein glycosylation [25]. Aberrant glycosylation is yet another feature expressed in cancer cells [26]. CRC metabolic model used in our study, was able to capture the alterations of certain enzymes catalyzing amino sugar metabolism in HBP. N-Acetylglucosamine Kinase ('ACGAMK') catalyzed phosphorylation reaction, which generates UDP-GlcNAc from N-Acetylglucosamine [27] showed increase in flux in the CRC model. Moreover, enzyme catalyzed reactions catalyzed by UDP-N-Acetyl-D-Glucosamine 2-Epimerase (Hydrolysis) and UDP-N-Acetylglucosamine 4-Epimerase, which are involved in sialic acid synthesis, also showed increased flux. Sialic acid is of major relevance in pathways involving cellular adhesion, cellular communication and signal transduction, and its deficiency has been linked to cancer and inflammatory disease [28].

9. Eicosanoid metabolism:

Eicosanoids (a class of bioactive lipids) are known to play a prominent role in carcinogenesis and metastatic processes [29]. Arachidonic acid is metabolized to generate eicosanoids through cyclooxygenase, lipoxygenase and P450 epoxygenase pathways [29]. Arachidonate 5-Lipoxygenase (ALOX 5) catalyzes the deoxygenation of arachidonic acid to initiate the synthesis of leukotrienes and is an important therapeutic target for various inflammatory diseases, including cancer [30]. For instance, the expression of ALOX 5 is reported to be upregulated in colon cancer cells [31]. An increased flux was observed through ALOX 5 reaction in CRC-microbe integrated model.

In addition to the predictions in line with literature-based information, the FSr analysis of models also had a few contradictory findings. These findings are discussed below:

1. Rewired fatty acid biosynthetic pathway:

In general, the fatty acid synthesis pathways showed increased fluxes in CRC and CRC-microbe metabolic models, thereby supporting increased cancer growth. However, the CRC-microbe model, when compared with the colon-microbe metabolism, showed reduced flux (decrease by 50%) in certain reactions involved in fatty acid synthesis, like, palmitoyl Coenzyme A hydrolase (EC:3.1.2.2) catalyzed reaction. This reduction in flux could be attributed to metabolic re-wiring to compensate for the other metabolic pathways that have been significantly upregulated in the metabolic models.

2. Altered TCA metabolism:

The TCA cycle comprises of many enzyme-catalyzed reactions, succinate dehydrogenase (SDH) being one of them. SDH catalyzes oxidation of succinate to generate fumarate. Also known as complex II, SDH is important in TCA cycle and electron transport chain. The inactivation of SDH results in succinate accumulation, thereby promoting tumorigenesis [32]. However, this SDH catalyzed reaction showed increased flux values in the CRC-microbe and CRC models.

3. Reduced flux through Prostaglandin I_2 Synthase (PTGIS) in colon cancer:

Prostanoids (prostaglandins, prostacyclin etc.) are a class of eicosanoids associated with the initiation and metastases of various cancer types like lung, breast, and endometrial cancers, as well as inhibition of melanoma [33]. Of the various types of prostaglandins (PGE_2, PGD_2, TXA_2), PGI_2 (also known as prostacyclin) has shown anti-metastatic properties in some studies surrounding murine tumorigenesis models [29]. However, the role of PTGIS in colorectal cancer is controversial, as some studies have stated that in case of chemical induced colon cancer in mice (with genetically

deleted PGIS), an increase in aberrant crypt formation was observed at early stages of carcinogenesis [34]. On the contrary, PTGIS upregulation has been implicated in colorectal cancer growth by preventing apoptosis [35]. Furthermore, recent studies have reported overexpression of PTGIS in cases of colon metastatic tumors in liver [36]. But the modeling results in cases of CRC and CRC-microbe integrated models have reported decreased flux through the PTGIS reaction.

Moreover, to uncover potent novel therapeutic metabolites with anticancer potential, shadow price analysis was used as a computational tool. Shadow price analysis can be used to capture any flux inconsistencies that are induced in a metabolic objective function (such as the maximization of biomass function), upon an increase or decrease in the fluxes associated with the metabolites participating in the individual reaction [37]. Hence, the shadow prices can mathematically analyze the sensitivity of the objective function of a linear program upon perturbation of individual constraints.

Shadow prices were analyzed for the biomass objective function ('biomass_reaction'). Metabolites from myo-inositol and inositol metabolism and their corresponding derivatives (i.e., 34 in total) resulted in positive shadow prices. Any changes in concentrations of these metabolites predicted by the shadow price analysis can deregulate the flux through the maximized biomass objective function. In other words, a positive shadow price of a metabolite indicates that biomass yield (flux) tends to decrease upon introducing any more concentration of that metabolite in the system. As far as the CRC model predictions are concerned, literature studies have emphasized on the anticancer potential of inositol and myo-inositol, wherein, inositol phosphates displayed inhibitory effects on cancer cells by causing cell cycle arrest at G1 phase of DNA synthesis [38].

Similarly, shadow price analysis was carried out for the healthy colon model to examine any overlap in the metabolites between CRC and healthy colon. For the colon model, 19 metabolites displayed positive shadow prices. Interestingly, most of them were lysine and lysine-derived metabolites, and there were no overlapping metabolites (like inositol or myo-inositol and their derivatives) between CRC and healthy colon model, with respect to shadow price analysis.

Since there were no overlapping metabolites between CRC and healthy colon model, inositol and its derivatives can be beneficial in treating/killing cancer cells, without posing any effects on healthy colon cells, as the growth (biomass) of healthy colon cells is independent of the changing concentrations of inositol and its derivatives. Thus, this analysis ensured the credibility of the metabolic models by highlighting the metabolites possessing anticancer activity.

Another important analysis performed on the CRC and colon models was flux enrichment analysis (FEA) to determine if the subset of enriched reactions (affected in colon v/s CRC) from different subsystems were contributing to the model structure in a statistically significant manner. The reactions (with FSr values in defined range, Figure 6a) belonging to different pathways like fatty acid oxidation and synthesis, amino acid metabolism, purine synthesis, folate, and sphingolipid metabolism were subjected to this analysis. The results predicted that the enriched reaction sets from both CRC and colon models were a part of a particular subsystem in a statistically significant manner. Supplementary Figure 7 tabulates the enriched reaction set information and their corresponding P-values:

Supplementary Figure 7: Flux enrichment analysis for statistical analysis of colon and CRC models

Colon					CRC				
'P-value'	'Adjusted P-value'	'Group'	'Enriched set size'	'Total set size'	'P-value'	'Adjusted P-value'	'Group'	'Enriched set size'	'Total set size'
0.005	0.005	'Fatty acid oxidation'	7	880	0.003	0.003	'Fatty acid oxidation'	7	915
3.66E-06	3.66E-06	'Fatty acid synthesis'	6	232	2.26E-06	2.26E-06	'Fatty acid synthesis'	6	239
6.72E-08	4.03E-07	'Tyrosine metabolism'	5	70	3.26E-05	0.00022816	'Tyrosine metabolism'	3	67
2.12E-08	2.12E-08	'Folate metabolism'	5	56	1.34E-08	1.34E-08	'Folate metabolism'	5	57
1.91E-18	1.91E-18	'Purine synthesis'	8	15	7.89E-19	7.89E-19	'Purine synthesis'	8	15
2.23E-14	4.45E-14	'Sphingolipid metabolism'	11	111	1.97E-15	3.95E-15	'Sphingolipid metabolism'	11	100

Since diet plays a crucial role in the initiation and progression of CRC, the model exchanges were constrained as per the Western diet (high protein, high fat content) and high fiber diet components [39]. The composition and uptake rates of these two diets' components have been tabulated in Supplementary Tables 3 and 4.

In order to monitor the cancer cell growth upon exposure to the Western and high fiber diets, the flux through CRC biomass reaction was computed in each case. On comparing the growth (flux) with DMEM constrained model, there was no change observed in the biomass flux (0.0223 mmol/g-DW/h) under the test dietary regime. Furthermore, to assess any significant changes in the overall metabolism of the model when constrained to these two diets, FVA was carried out for CRC. There too, no significant changes in the minimum/maximum flux values were captured in the sensitive reactions as identified by the FSr analysis for DMEM constrained models. Thus, CRC metabolism was not influenced by the change in diets as such.

Supplementary Table 3: The exchanges present in CRC and the respective lower bounds for constraining the model as per Western diet.

Substrate Exchanges	Lower Bounds		Substrate Exchanges	Lower Bounds
'EX_glc_D[e]'	-0.14899		'EX_amet[e]'	-1
'EX_gal[e]'	-0.14899		'EX_amp[e]'	-1
'EX_man[e]'	-0.14899		'EX_btn[e]'	-1
'EX_fuc_L[e]'	-0.14899		'EX_cgly[e]'	-1
'EX_glcn[e]'	-0.14899		'EX_chol[e]'	-1
'EX_arab_L[e]'	-0.17878		'EX_cit[e]'	-1
'EX_drib[e]'	-0.17878		'EX_csn[e]'	-1
'EX_rib_D[e]'	-0.17878		'EX_cytd[e]'	-1
'EX_xyl_D[e]'	-0.17878		'EX_dad_2[e]'	-1
'EX_oxa[e]'	-0.44696		'EX_dcyt[e]'	-1
'EX_lcts[e]'	-0.074493		'EX_dgsn[e]'	-1
'EX_malt[e]'	-0.074493		'EX_etoh[e]'	-1
'EX_tre[e]'	-0.074493		'EX_fald[e]'	-1
'EX_strch1[e]'	-0.25734		'EX_fe2[e]'	-1
'EX_arachd[e]'	-3.33E-03		'EX_fe3[e]'	-1
'EX_chsterol[e]'	-0.004958		'EX_fol[e]'	-1
'EX_glyc[e]'	-1.7997		'EX_for[e]'	-1
'EX_hdca[e]'	-0.39637		'EX_fum[e]'	-1
'EX_hdcea[e]'	-0.036517		'EX_gam[e]'	-1
'EX_lnlnca[e]'	-0.017565		'EX_glyc3p[e]'	-1
'EX_lnlncg[e]'	-0.017565		'EX_gsn[e]'	-1
'EX_ocdca[e]'	-0.16928		'EX_gthox[e]'	-1
'EX_ocdcea[e]'	-0.68144		'EX_gthrd[e]'	-1
'EX_octa[e]'	-0.012943		'EX_gua[e]'	-1
'EX_ttdca[e]'	-0.068676		'EX_h[e]'	-1
'EX_ala_L[e]'	-1		'EX_hom_L[e]'	-1
'EX_cys_L[e]'	-1		'EX_hxan[e]'	-1
'EX_ser_L[e]'	-1		'EX_ins[e]'	-1
'EX_arg_L[e]'	-0.15		'EX_k[e]'	-1
'EX_his_L[e]'	-0.15		'EX_lac_L[e]'	-1
'EX_ile_L[e]'	-0.15		'EX_mal_L[e]'	-1
'EX_leu_L[e]'	-0.15		'EX_mqn7[e]'	-1
'EX_lys_L[e]'	-0.15		'EX_mqn8[e]'	-1
'EX_asn_L[e]'	-0.225		'EX_na1[e]'	-1
'EX_asp_L[e]'	-0.225		'EX_ncam[e]'	-1
'EX_thr_L[e]'	-0.225		'EX_no2[e]'	-1
'EX_glu_L[e]'	-0.18		'EX_orn[e]'	-1
'EX_met_L[e]'	-0.18		'EX_pheme[e]'	-1
'EX_gln_L[e]'	-0.18		'EX_pi[e]'	-1
'EX_pro_L[e]'	-0.18		'EX_pnto_R[e]'	-1
'EX_val_L[e]'	-0.18		'EX_ptrc[e]'	-1
'EX_phe_L[e]'	-1		'EX_pydam[e]'	-1
'EX_tyr_L[e]'	-1		'EX_pydx[e]'	-1
'EX_gly[e]'	-0.45		'EX_pydx5p[e]'	-1
'EX_trp_L[e]'	-0.08182		'EX_pydxn[e]'	-1
'EX_2obut[e]'	-1		'EX_ribflv[e]'	-1
'EX_ac[e]'	-1		'EX_so4[e]'	-1
'EX_acald[e]'	-1		'EX_spmd[e]'	-1
'EX_acgam[e]'	-1		'EX_thm[e]'	-1
'EX_acmana[e]'	-1		'EX_thymd[e]'	-1
'EX_acnam[e]'	-1		'EX_ura[e]'	-1
'EX_ade[e]'	-1		'EX_uri[e]'	-1
'EX_adn[e]'	-1		'EX_xan[e]'	-1
'EX_akg[e]'	-1		'EX_meoh[e]'	-10
'EX_ala_D[e]'	-1		'EX_h2o[e]'	-10

Supplementary Table 4: The exchanges present in CRC and the respective lower bounds for constraining the model as per high fiber diet.

Substrate Exchanges	Lower Bounds		Substrate Exchanges	Lower Bounds
EX_glc_D[e]	-0.03947		EX_retinol[e]	-1
EX_gal[e]	-0.03947		EX_thf[e]	-1
EX_man[e]	-0.03947		EX_2obut[e]	-1
EX_fuc_L[e]	-0.03947		EX_ac[e]	-1
EX_glcn[e]	-0.03947		EX_acgam[e]	-1
EX_arab_L[e]	-0.04737		EX_acmana[e]	-1
EX_drib[e]	-0.04737		EX_acnam[e]	-1
EX_rib_D[e]	-0.04737		EX_ade[e]	-1
EX_xyl_D[e]	-0.04737		EX_adn[e]	-1
EX_oxa[e]	-0.11842		EX_ala_D[e]	-1
EX_lcts[e]	-0.01974		EX_amp[e]	-1
EX_malt[e]	-0.01974		EX_btn[e]	-1
EX_tre[e]	-0.01974		EX_cgly[e]	-1
EX_strch1[e]	-0.06818		EX_chol[e]	-1
EX_arachd[e]	-0.001664		EX_cit[e]	-1
EX_chsterol[e]	-0.002479		EX_csn[e]	-1
EX_glyc[e]	-0.89983		EX_dad_2[e]	-1
EX_hdca[e]	-0.19819		EX_dcyt[e]	-1
EX_hdcea[e]	-0.018258		EX_dgsn[e]	-1
EX_lnlnca[e]	-0.008783		EX_fald[e]	-1
EX_lnlncg[e]	-0.008783		EX_fe2[e]	-1
EX_ocdca[e]	-0.084641		EX_fe3[e]	-1
EX_ocdcea[e]	-0.34072		EX_fol[e]	-1
EX_octa[e]	-0.006471		EX_for[e]	-1
EX_ttdca[e]	-0.034338		EX_fum[e]	-1
EX_ala_L[e]	-1		EX_gam[e]	-1
EX_cys_L[e]	-1		EX_glyc3p[e]	-1
EX_ser_L[e]	-1		EX_gthox[e]	-1
EX_arg_L[e]	-0.15		EX_gthrd[e]	-1
EX_his_L[e]	-0.15		EX_gua[e]	-1
EX_ile_L[e]	-0.15		EX_h[e]	-1
EX_leu_L[e]	-0.15		EX_hxan[e]	-1
EX_lys_L[e]	-0.15		EX_k[e]	-1
EX_asn_L[e]	-0.225		EX_mqn7[e]	-1
EX_asp_L[e]	-0.225		EX_mqn8[e]	-1
EX_thr_L[e]	-0.225		EX_na1[e]	-1
EX_glu_L[e]	-0.18		EX_ncam[e]	-1
EX_met_L[e]	-0.18		EX_no2[e]	-1
EX_gln_L[e]	-0.18		EX_no2[e]	-1
EX_pro_L[e]	-0.18		EX_orn[e]	-1
EX_val_L[e]	-0.18		EX_pheme[e]	-1
EX_phe_L[e]	-1		EX_pi[e]	-1
EX_tyr_L[e]	-1		EX_pnto_R[e]	-1
EX_gly[e]	-0.45		EX_ptrc[e]	-1
EX_trp_L[e]	-0.08182		EX_pydam[e]	-1
EX_5aop[e]	-1		EX_pydx[e]	-1
EX_acald[e]	-1		EX_pydx5p[e]	-1
EX_akg[e]	-1		EX_pydxn[e]	-1
EX_amet[e]	-1		EX_ribflv[e]	-1
EX_anth[e]	-1		EX_so4[e]	-1
EX_avite1[e]	-1		EX_spmd[e]	-1
EX_cytd[e]	-1		EX_thm[e]	-1
EX_etoh[e]	-1		EX_thymd[e]	-1
EX_glyleu[e]	-2		EX_ura[e]	-1
EX_gsn[e]	-1		EX_uri[e]	-1
EX_hom_L[e]	-1		EX_xan[e]	-1
EX_ins[e]	-1		EX_meoh[e]	-10
EX_lac_L[e]	-1		EX_h2o[e]	-10
EX_mal_L[e]	-1			

Supplementary Data

Supplementary Data 1
Excel sheet 1-A: MRS medium composition
Excel sheets 1-B: MRS medium constrained *E. durans* metabolic model
Excel sheets 1-C: MRS medium constrained ROS expanded *E. durans* metabolic model

Supplementary Data 2
Excel sheet 2-A: DMEM composition
Excel sheets 2-B: DMEM constrained healthy colon metabolic model
Excel sheets 2-C: DMEM constrained healthy colon metabolic model (with sink reactions)
Excel sheets 2-D: DMEM constrained CRC metabolic model

Supplementary Data 3
Excel sheets 3-A: Colon-*E. durans* integrated metabolic model
Excel sheets 3-B: CRC-*E. durans* integrated metabolic model

Supplementary Note

%% Codes used %%

%% Flux Variability Analysis for a model %%

```
[minFlux,maxFlux]=fluxVariability(model,0);
```

%% minNorm analysis of ROS-E.durans model %%

```
model = changeObjective (model, 'RXN1');
% RXN1 is the reaction with FSr in the range: 0.8>FSr>2 %
% RXN: 'DHFOR2', 'DHFR', 'FTHFL', 'MTHFC', 'GLYCL', 'r0792'  %
FBA=optimizeCbModel (model, 'max', 'one');
% Access FVA.v to see the relevant affected reactions %
```

%% Shadow price analysis for a model %%

```
% Please refer to Supplementary Discussion, section 1D) %

model=changeObjective(model,'biomass_reaction'); %for CRC model
FBA=optimizeCbModel(model,'max');
% Access FVA.y to see the Shadow Prices of Metabolites
```

Supplementary References

1. Bhakta-Guha, D. & Efferth, T. Hormesis: Decoding Two Sides of the Same Coin. *Pharmaceuticals* **8**, 865–883 (2015).
2. Moffatt, B. A. & Ashihara, H. Purine and Pyrimidine Nucleotide Synthesis and Metabolism. *Arabidopsis Book* **1**, (2002).
3. Khutornenko, A. A., Dalina, A. A., Chernyak, B. V., Chumakov, P. M. & Evstafieva, A. G. The Role of Dihydroorotate Dehydrogenase in Apoptosis Induction in Response to Inhibition of the Mitochondrial Respiratory Chain Complex III. *Acta Naturae* **6**, 69–75 (2014).
4. Sykes, D. B. The emergence of dihydroorotate dehydrogenase (DHODH) as a therapeutic target in acute myeloid leukemia. *Expert Opin Ther Targets* **22**, 893–898 (2018).
5. Tirinato, L. *et al.* An Overview of Lipid Droplets in Cancer and Cancer Stem Cells. *Stem Cells Int* **2017**, (2017).
6. Zaugg, K. *et al.* Carnitine palmitoyltransferase 1C promotes cell survival and tumor growth under conditions of metabolic stress. *Genes Dev* **25**, 1041–1051 (2011).
7. Nakajima, Y. *et al.* Three-dimensional structure of the flavoenzyme acyl-CoA oxidase-II from rat liver, the peroxisomal counterpart of mitochondrial acyl-CoA dehydrogenase. *J. Biochem.* **131**, 365–374 (2002).
8. Agnihotri, G. & Liu, H. Enoyl-CoA hydratase. reaction, mechanism, and inhibition. *Bioorg. Med. Chem.* **11**, 9–20 (2003).
9. Zhao, Q.-M., Kuang, F., Wu, H. & Zhang, Y.-H. Attenuation of enoyl coenzyme A hydratase 1 expression in colorectal cancer cells using small interfering RNA inhibits cell proliferation and migration. *Molecular Medicine Reports* **12**, 470–474 (2015).
10. Chang, W. C., Chapkin, R. S. & Lupton, J. R. Predictive value of proliferation, differentiation and apoptosis as intermediate markers for colon tumorigenesis. *Carcinogenesis* **18**, 721–730 (1997).
11. Berg, J. M., Tymoczko, J. L. & Stryer, L. Fatty Acids Are Synthesized and Degraded by Different Pathways. *Biochemistry. 5th edition* (2002).
12. Adeva-Andany, M. M., López-Maside, L., Donapetry-García, C., Fernández-Fernández, C. & Sixto-Leal, C. Enzymes involved in branched-chain amino acid metabolism in humans. *Amino Acids* **49**, 1005–1028 (2017).
13. Lynch, C. J. & Adams, S. H. Branched-chain amino acids in metabolic signalling and insulin resistance. *Nat Rev Endocrinol* **10**, 723–736 (2014).
14. Gwaltney-Brant, S. M. Chapter 7 - Nutraceuticals in Hepatic Diseases. in *Nutraceuticals* (ed. Gupta, R. C.) 87–99 (Academic Press, 2016). doi:10.1016/B978-0-12-802147-7.00007-3.
15. Corbet, C. Stem Cell Metabolism in Cancer and Healthy Tissues: Pyruvate in the Limelight. *Front Pharmacol* **8**, (2018).
16. Yue, W. *et al.* Effects of estrogen on breast cancer development: role of estrogen receptor independent mechanisms. *Int J Cancer* **127**, 1748–1757 (2010).

17. Rawłuszko-Wieczorek, A. A. *et al.* Significance of intratissue estrogen concentration coupled with estrogen receptors levels in colorectal cancer prognosis. *Oncotarget* **8**, 115546–115560 (2017).
18. Day, J. M. *et al.* The development of steroid sulfatase inhibitors for hormone-dependent cancer therapy. *Ann. N. Y. Acad. Sci.* **1155**, 80–87 (2009).
19. He, W., Gauri, M., Li, T., Wang, R. & Lin, S.-X. Current knowledge of the multifunctional 17β-hydroxysteroid dehydrogenase type 1 (HSD17B1). *Gene* **588**, 54–61 (2016).
20. Africander, D. & Storbeck, K.-H. Steroid metabolism in breast cancer: Where are we and what are we missing? *Molecular and Cellular Endocrinology* **466**, 86–97 (2018).
21. Mullen, P. J., Yu, R., Longo, J., Archer, M. C. & Penn, L. Z. The interplay between cell signalling and the mevalonate pathway in cancer. *Nat. Rev. Cancer* **16**, 718–731 (2016).
22. Swanson, K. M. & Hohl, R. J. Anti-cancer therapy: targeting the mevalonate pathway. *Curr Cancer Drug Targets* **6**, 15–37 (2006).
23. Jung, E. J., Chung, K. H. & Kim, and C. W. Identification of simvastatin-regulated targets associated with JNK activation in DU145 human prostate cancer cell death signaling. *BMB Reports* **50**, 466–471 (2017).
24. Sharon, C. *et al.* Inhibition of insulin-like growth factor receptor/AKT/mammalian target of rapamycin axis targets colorectal cancer stem cells by attenuating mevalonate-isoprenoid pathway in vitro and in vivo. *Oncotarget* **6**, 15332–15347 (2015).
25. de Queiroz, R. M. *et al.* Hexosamine Biosynthetic Pathway and Glycosylation Regulate Cell Migration in Melanoma Cells. *Front Oncol* **9**, (2019).
26. Akella, N. M., Ciraku, L. & Reginato, M. J. Fueling the fire: emerging role of the hexosamine biosynthetic pathway in cancer. *BMC Biol* **17**, 52 (2019).
27. Furo, K., Nozaki, M., Murashige, H. & Sato, Y. Identification of an N-acetylglucosamine kinase essential for UDP-N-acetylglucosamine salvage synthesis in Arabidopsis. *FEBS Letters* **589**, 3258–3262 (2015).
28. Wang, B. & Brand-Miller, J. The role and potential of sialic acid in human nutrition. *Eur J Clin Nutr* **57**, 1351–1369 (2003).
29. Gomes, R. N., Felipe da Costa, S. & Colquhoun, A. Eicosanoids and cancer. *Clinics (Sao Paulo)* **73**, (2018).
30. Anwar, Y., Sabir, J. S. M., Qureshi, M. I. & Saini, K. S. 5-lipoxygenase: a promising drug target against inflammatory diseases-biochemical and pharmacological regulation. *Curr Drug Targets* **15**, 410–422 (2014).
31. Wang, Y. *et al.* Eicosanoid signaling in carcinogenesis of colorectal cancer. *Cancer Metastasis Rev* **37**, 257–267 (2018).
32. Moosavi, B., Zhu, X., Yang, W.-C. & Yang, G.-F. Molecular pathogenesis of tumorigenesis caused by succinate dehydrogenase defect. *European Journal of Cell Biology* 151057 (2019) doi:10.1016/j.ejcb.2019.151057.

33. Tennis, M. A., Vanscoyk, M., Keith, R. L. & Winn, R. A. The role of prostacyclin in lung cancer. *Transl Res* **155**, 57–61 (2010).
34. Sasaki, Y. *et al.* Role of prostacyclin synthase in carcinogenesis. *Prostaglandins & Other Lipid Mediators* **133**, 49–52 (2017).
35. Cutler, N. S. *et al.* Stromal production of prostacyclin confers an antiapoptotic effect to colonic epithelial cells. *Cancer Res.* **63**, 1748–1751 (2003).
36. Lichao, S. *et al.* Overexpression of PTGIS could predict liver metastasis and is correlated with poor prognosis in colon cancer patients. *Pathol. Oncol. Res.* **18**, 563–569 (2012).
37. Sajitz-Hermstein, M. & Nikoloski, Z. Multi-objective shadow prices point at principles of metabolic regulation. *Biosystems* **146**, 91–101 (2016).
38. Singh, R. P., Agarwal, C. & Agarwal, R. Inositol hexaphosphate inhibits growth, and induces G1 arrest and apoptotic death of prostate carcinoma DU145 cells: modulation of CDKI-CDK-cyclin and pRb-related protein-E2F complexes. *Carcinogenesis* **24**, 555–563 (2003).
39. Magnúsdóttir, S. *et al.* Generation of genome-scale metabolic reconstructions for 773 members of the human gut microbiota. *Nat. Biotechnol.* **35**, 81–89 (2017).
